# Optimization design and experiment of key components of mountain pendulum-lever cam type hole seeders based on DEM-MBD coupling simulation

**DOI:** 10.1371/journal.pone.0313285

**Published:** 2025-03-14

**Authors:** Lijun Zhao, Wenke Yin, Bin Yang, Xin Hu, Chuandong Liu, Zihuan Li, Lian Gong, Qiang Li, Bin Li, Shang Li

**Affiliations:** 1 Intelligent Manufacturing Engineering College of Chongqing Wenli University, Chongqing, China; 2 School of Mechanical Engineering, Jiamusi University, Jiamus, China; 3 Chongqing General Station of Agricultural Mechanization Technology Promotion, Chongqing, China; Federal University of Technology—Parana, BRAZIL

## Abstract

In response to issues such as high miss-seeding rates and uneven seed grain distribution during the operation of the pendulum-lever camshaft hole seeders under the compound planting mode of corn and soybeans in hilly mountainous areas, a method for optimizing the hole seeders by adjusting movable pendulum-lever angle and the number of cam roller groups is proposed. By analyzing the motion process and mechanism of hole formation of the hole-forming device, it was possible to elucidate the influence of the movable pendulum-lever angle and cam roller group number on the improvement of seeding quality. Based on the DEM-MBD coupled simulation, single-factor simulation experiments were conducted using the hole seeders shaft speed, movable pendulum- lever angle, and cam roller group number as test factors, with the seed grain qualification rate, reseeding rate, and miss-seeding rate as test indicators. A three-factor, three-level orthogonal rotation combination simulation experiment was designed to derive a mathematical model of the relationship between test factors and indicators. Data analysis was performed using Design-Expert 13 soft-ware to optimize the regression model for multiple objectives and obtain the optimal parameter combination. The simulation test results indicate that when the hole seeder shaft speeds were 47.43 r/min and 48.09 r/min, the movable pendulum- lever angles were 100.23° and 101.70°, and the number of rollers in the cam group were 2.81 and 2.95,the qualified rates of corn and soybean seeding were 95.19% and 96.07%. The reseeding rates were 3.58% and 2.35%, while the missed seeding rates were 1.23% and 1.58%. The field test results indicate that under the optimal parameter combination, the relative errors of the qualification rate, the reseeding rate, and the missed seeding rate between the simulation tests and the field tests were 0.4% and 0.13%, 1.17% and 0.36%, and 1.57% and 0.23%. This serves to validate the accuracy of the coupled simulation model, and the research findings can provide theoretical support and a point of reference for the design and performance optimization of pendulum- lever cam-type hole seeders in hilly and mountainous areas.

## 1. Introduction

Corn and soybean as China’s important grain, oil and feed dual-use crops, the conflict over land is prominent [1–3]. Hilly and mountainous areas have poor natural conditions, complex topography and geomorphology, fine and messy plots of land, irregular shape, and as the compound planting area of corn and soybeans continues to expand, many precision hole seeders can not meet the agronomic requirements of sowing under these complex conditions [4,5], so there is an urgent need to solve the operational performance and sowing efficiency of the hole seeder in the mountainous operating environment, which is of great importance in improving the yield of corn and soybeans in China, as well as in guaranteeing food security. It is of great significance in improving corn and soybean yields and ensuring food security in China.

At present, mechanical seed dispensers have become the most widely used seed dispensers in hilly and mountainous conditions due to their simple structure, high reliability, and the ability to be matched with small and medium-sized agricultural implements [6]. Sun Wei et al. [7] mainly controlled the duckbill hole-former by studying the speed compensation mechanism, cam and crank combination mechanism, and parallel four-bar mechanism, so as to attenuate the horizontal movement during the seeding period of soil entry-exit to alleviate the leakage of seeding caused by the phenomena of tearing, picking, and misalignment of the film; Shi Linrong et al. [8] designed a hole-former forward speed compensation mechanism, in which a rotating guide rod mechanism drives a parallel four-bar mechanism to compensate for the forward speed of the implement to reduce the horizontal displacement difference between the hole-former’s entry into the soil for planting and its exit from the soil. However, these studies have neglected the effect of hole-forming devices on seeding results. Speeding up the operation of a hole seeders results in shorter planting times, which may result in the hole-forming planter not being able to distribute seed successfully or seed not being distributed in a timely manner, leading to seed clogging and damage. Research on the effect of hole-forming mechanisms on seeding outcomes during planter operation is still limited. Han Changjie et al. [9] analyzed the motion mechanism of the hole-former and established the parametric equation of the planting hole according to the characteristics of the dryland transplanting machinery, and concluded that the theoretical depth of the hole-former is the main factor affecting the longitudinal length of the upper part of the hole opening; LIU et al. [10] used a five-link mechanism and a slider mechanism to control the action of the duckbill planter, and solved the problems of large transplanting holes in the membrane, low uprightness, and the hole opening of the film breakage, etc.; Wang Yicai et al. [11] designed a vertical emergence and entry hole seeders mechanism, which utilizes a parallel four-link mechanism to ensure vertical movement of the hole seeders during emergence and entry; Li Yajun et al. [12] used the motion plug-in of SOLIDWORKS to simulate and analyze the trajectory of the duckbill of the hole seeding wheel in order to reduce the error of the hole seeding wheel on the tearing and picking of the mulch; Zhao Jiantuo et al. [13] carried out the cam design of the duckbill forced-opening mechanism and tested the pendulum angle, angular velocity, and angular acceleration of the duckbill in ADAMS using the method of generating solids from relative trajectory curves. However, this kind of research mainly improves the seeding performance by optimizing the linkage mechanism or improving the seeding method. Whether the seed is completely released from the duckbill into the soil is also particularly critical. If the hole-former does not open and close completely during the seeding process, resulting in soil carryover or soil entrapment, then it is easy to produce leakage, reseeding, and reduced seeding accuracy. It is particularly important to study the impact of hole planter and its hole forming effect on seeding performance.

In summary, in order to achieve the goals of high quality demand of mechanical hole seeders into holes under the operating environment of sticky and heavy soil in hilly mountainous areas, and to reduce the rebroadcast rate and miss seeding rate, this paper adopts DEM-MBD coupled simulation to optimize the design of the pendulum-lever cam-type hole seeders according to the agronomic requirements of Corn and Soya bean compound planting in hilly mountainous areas. Focusing on the principle of hole seeders hole formation, the optimal combination of parameters of the hole planter hole formation key components of the two key elements of the research, to obtain an increase in the number of holes qualified rate and reduce miss seeding rate of the sowing process, in order to provide some theoretical support and reference to the hilly mountainous areas of soybean and corn belt composite seeding machine.

## 2. Materials and methods

### 2.1. The agronomic requirements for corn and soybean intercropping

**Fig 1:**
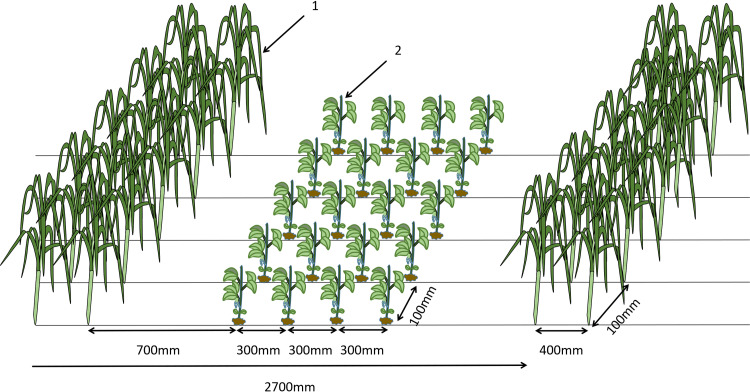
A diagrammatic representation of the ‘4+2’ pattern for the intercropping of soybean and corn. 1. Corn plant, 2. Soybean plant.

Currently, there are various strip intercropping patterns. In response to the characteristics of the humid climate, sticky soil, and small, scattered plots in the hilly areas of Southwest China, aiming to improve land utilization efficiency, a whole-field design approach has been adopted. This involves significantly reducing the spacing between corn plants to 100 mm within rows spaced 400 mm apart, achieving a planting density for pure stands. For soybeans, the spacing has been moderately reduced to 100 mm within rows spaced 300 mm apart, achieving a planting density of over 70% of the pure stand density. The primary planting pattern, illustrated in Fig 1, is the “4+2” pattern, in which soybeans and corn are intercropped with four rows of soybeans and two rows of corn [14–16].

### 2.2. Overall structure and working principle

#### 2.2.1. Overall structure.

**Fig 2:**
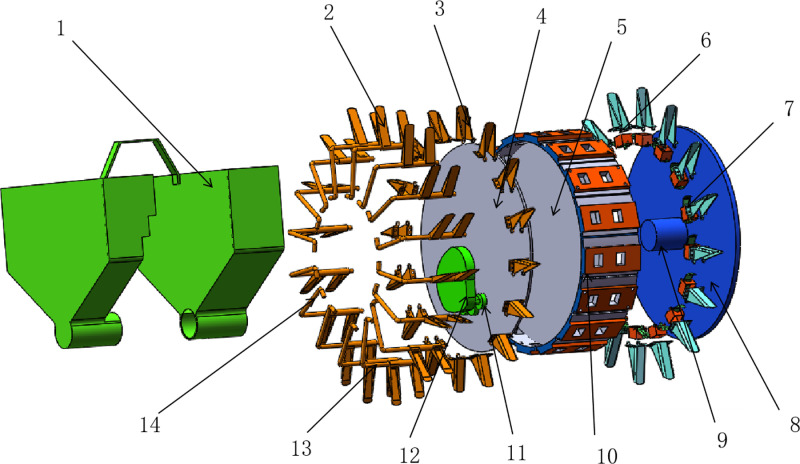
Diagram of the internal structure of a swinging arm cam-type seed planter. 1. Fertilizer box, 2. Moving duck bill, 3. Fixing duck bill, 4. Side plate, 5. Divider, 6. Moving duck bill, 7. Seed scoop, 8. Side plate, 9. Burrow planter shaft, 10. Burrow planter shel, 11. Roller, 12. Cam group mechanism, 13. Reset spring, 14. Movable pendulum rod.

The swinging arm cam-type seed planter is comprised of a multitude of components, including fixed and moving duckbills, reset springs, swinging arms, cam mechanism groups, seed and fertilizer boxes, seed scoops, hole planter shafts, and side plates. A schematic diagram of the structure is provided in Fig 2.

#### 2.2.2. Working principle.

**Fig 3:**
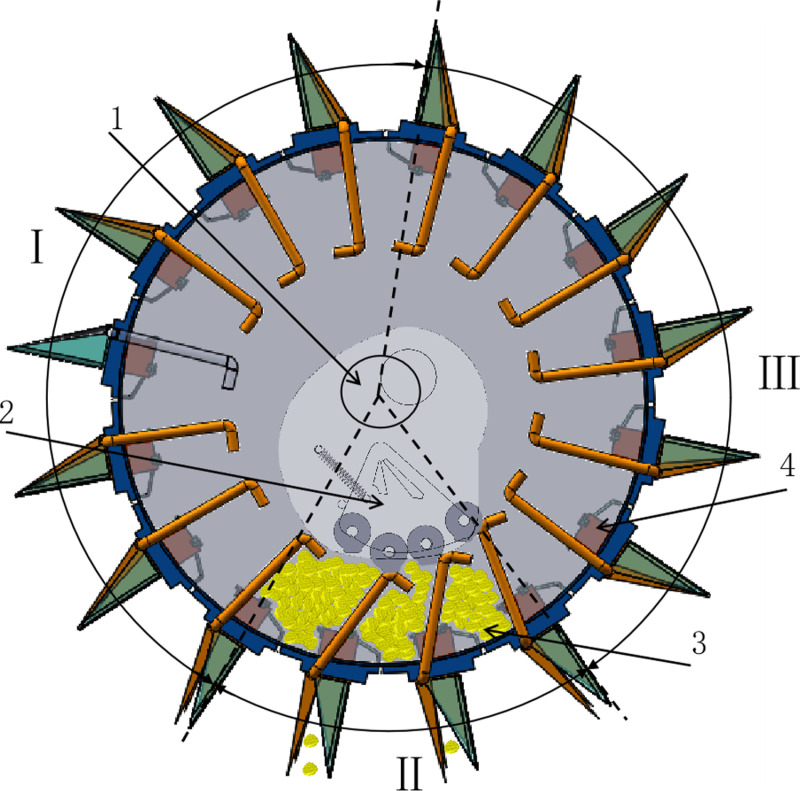
The working process of the dibbler seeder. I. Filling area, II. Seeding area, III. Carrying area, 1. Shaft of burrow planter, 2. Cam group mechanism, 3. Seed, 4. Take seed blocks.

Manual placement of seeds into the seed box forms a heap. Under the influence of mutual forces and gravity, seed groups dynamically flow within the seed-carrying area of the seed planter shell. Seeds in the filling area enter the seed-taking chamber relying on gravity and inter-seed forces. When the duckbill seeder rotates to a specific position, seeds fall into the cavity of the seeder under the force of gravity. At this moment, the swinging arm slides around the outer circle of the roller assembly on the cam, with the rollers acting on the upper end of the swinging arm to keep the duckbill open. Subsequently, the seeds drop into the soil, completing one planting cycle, as shown in Fig 3.

### 2.3. Key component design and analysis

The hole-forming device plays a vital role in the seeding process in hilly and mountainous areas, which is mainly composed of moving and fixed duckbill and pendulum- lever cam group mechanism. The opening angle and time of the hole-former are the key factors affecting the seeding performance of the hole-former. The opening angle is too large or the opening time is too long, which can lead to clogging of the soil transport and leakage of the seed [17,18]. Therefore, it is important to minimize the amount of soil disturbance caused by the hole former, while ensuring smooth seeding to maintain seeding efficiency [19,20].

#### 2.3.1. Design of the hole forming device.

**Fig 4:**
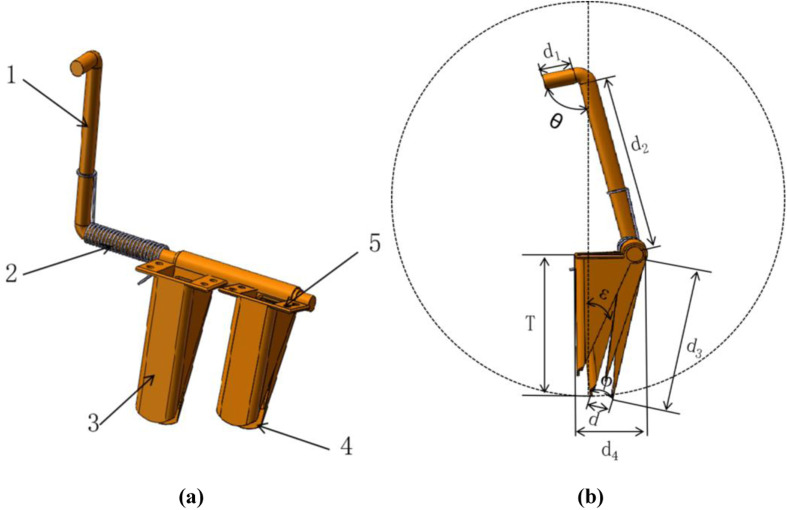
The hole forming device structure. (a) the hole forming device model drawing, (b) principle schematic. 1. Movable swing rod, 2. Reset spring, 3. Fixed beak, 4. Moving beak, 5. Fixed base.

The main shapes of hole-formers are conical and wedge-shaped, and studies have shown that conical hole-formers are more effective than wedge-shaped hole-formers in terms of hole formation and soil movement [21]. Taper the hole forming device mainly consists of a fixed duckbill, a fixed base, a movable pendulum- lever, a reset spring and a movable duckbill, as shown in Fig 4.

As shown in Fig 4b, to ensure that the seed drop is smooth and the hole forming device works without clogging and carrying soil, the hole forming device opening d satisfies the [22].


$$d=1.2�1.5L_{\max}$$ (1)

here, d is the hole forming device opening; Lmax is the maximum seed length size.

Substituting the data in Table 1 into equation (1) yields a range of the hole forming device openings from 11.81 to 16.77 mm.

**Table 1:** Corn and soybean triaxial dimensions.

Seed	Median triaxial size (Length × width × thickness)/mm	Maximum length dimension/mm	Maximum width size/mm	Maximum thickness dimension/mm
Corn	9.26 × 6.87 × 5.14	10.18	7.08	6.07
Soya bean	8.11 × 6.95 × 5.33	9.83	7.25	6.36

The length of the movable duckbill not only affects the seeding depth, but also affects the scraping action of the tip as the soil flows out. If the length of the movable duckbill is too short, it is difficult to get the hole forming into soil; if the length of the movable duckbill is too long, it is easy to carry the soil and even block the hole forming device, which affects the emergence of seedlings. The formula for the length of the moving duckbill is


$$d_{3}=\frac{T}{\cos\varepsilon }$$ (2)

here, d_3_ is movable duckbill length; T is the Sowing depth; cos ε is the angle between the hole-forming edge line of the fixed duckbill and the bottom and center lines of hole seeders housing.

According to the requirements of agronomic technology of corn and soybean composite planting [23], T was taken as 30 mm; Reference [24], ε = 30°, substituting into Eq. (2) yields d_3_ = 34.64 mm. Be sure After the hole forming device opening, there is a geometric relationship between the angle φ of the moving duck’s beak and the hole forming device opening.


$$\phi =2\sin^{-1}\left(\frac{d}{2d_{3}}\right)$$ (3)

here, φ is the rotational angle of the moving duckbill.

The rotation angle φ of the movable duckbill was thus determined to be 11.54 to 16.36°. Pendulum- lever d_2_ is the distance from the hole forming part to the cam group, 80 mm; in order to ensure the stability of the opening of the hole forming machine, the value of pendulum-lever d_1_ should be 15 mm larger than the radius of the roller of the cam group, and 30 mm smaller than the center distance between the two neighboring rollers, which is taken as d_1_ = 22 mm.

### 2.4. Motion analysis of the hole formers device

**Fig 5:**
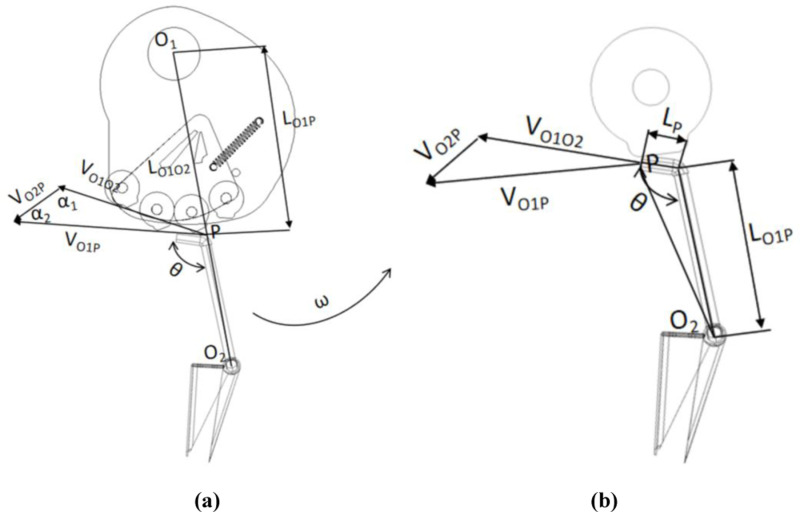
The hole formers and cam motion analysis. (a) Cam and pendulum-lever geometry relation, (b) Cam and pendulum-lever locomotor relations.

When the hole formers planter operation, the outer ring of the hole formers planter in the soil friction under the action of rolling forward, cam and frame solid connection relative to the ground flat, so the hole formers planter on the outer ring of the hole formers planter in the circular motion at the same time with the cam contact, at this time the hole formers planter to do the opening and closing movement. With the planter frame as a fixed coordinate system, using the reversal method to give the hole seeder and the actual direction of movement opposite, equal size of the angular velocity, at this time, the hole seeder outer ring is stationary, the cam group around the axis of the connection axis to do a uniform speed rotation, as shown in Fig 5.

Using the point-synthesis motion method to analyze the relationship between the cam and the movable rocker motion shown in Fig 5, where the point P is the contact point between the cam roller and the movable pendulum- lever, O_1_ is the center of rotation of the cam, and O_2_ is the center of rotation of the duckbill rocker, according to the geometrical relationships can be obtained.


$$\vec{V_{O1P}}=\vec{V_{O2P}}+\vec{V_{O1O2}}$$ (4)

here, V_O1P_ is the Absolute velocity of the movable pendulum at cam midpoint P; V_O2P_ is the rotational velocity of point P with respect to O_2_; V_O1O2_ is the cam and movable pendulum- lever engagement speed.

According to the sine theorem combined with the geometric relationship in Fig 5, it can be concluded that the triangular angles of the velocity vectors are respectively.


$$\sin\alpha_{1}=\frac{L_{O1O2}}{L_{O2P}}\sin(\omega t)$$ (5)

$$\cos\alpha_{2}=\frac{L_{O2}}{L_{O2P}}\sin\theta$$ (6)

Here, α_1_ is the angle between V_O2P_ and V_O1P_; α_2_ is the angle between V_O2P_ and V_O1O2_; θ is the Swing rod angle; L_O1O2_ is the distance between the center of rotation of the cam and the center of rotation of the movable pendulum- lever; L_O2P_ is the distance between the center of rotation of the movable pendulum- lever and the cam contact point; L_O2_ is the distance from center of rotation of movable pendulum- lever rod to turning point; ω is the Rotational angular velocity of the hole formers seeder.

Combine formula (4) to (6) to obtain


$$V_{O2P}=\frac{\sin(\alpha_{1}+\alpha_{2})}{\sin\alpha_{2}}V_{O1P}$$ (7)

According to the analytical process and equation (7), it can be seen that the form of motion of the hole formers is related to the pinch angles α_1_ and α_2_, that is, to the angle of the movable pendulum- lever and the rotational speed.

#### 2.4.1. Laws of motion of pendulum-lever and cams.

The functional relationship between the angular displacement turned by the pendulum rod and the pendulum rod pendulum angle determines the shape of the cam, and the pendulum rod pendulum angle is determined by the movable nozzle motion law, i.e., the relationship between the angular displacement turned by the pendulum rod and the pendulum angle of the movable nozzle is shown in Table 2:

**Table 2:** Pendulum-lever cam motion law.

	Angular displacement turned by the pendulum	Movable duckbill corner
Push phase	$0\leq \omega \leq \frac{\pi }{15}$	The swing increases to $\psi_{\max}$
Far Rest Phase	$\frac{\pi }{15}\leq \omega \leq \frac{19\pi }{90}$	Hold $\psi_{\max}$
Return Phase	$\frac{19\pi }{90}\leq \omega \leq \frac{5\pi }{18}$	Oscillation decreases to 0
Near Rest Phase	$\frac{5\pi }{18}\leq \omega \leq 2\pi$	Hold 0

According to the requirements of the work of the selected follower of the law of motion, from the working principle of the cavity seeder can be known duckbill forced to open the mechanism of the follower of the motion cycle for the rise of a stop a drop a stop, so the cam’s ascending and returning to select the pendulum line of motion law, to ensure that the pendulum rod pendulum angle, angular velocity, angular acceleration of the continuity of the pendulum rod, then it can be known that the cam angle of the pendulum angle of the cam and the movable beak pendulum angle of the thrust section of the motion of the function:


$$\psi =\psi_{\max}(\frac{\omega }{\varphi }-\frac{1}{2\pi }\sin\frac{2\pi }{\varphi }),\;0\leq \omega \leq \frac{\pi }{15}$$ (8)

**Fig 6:**
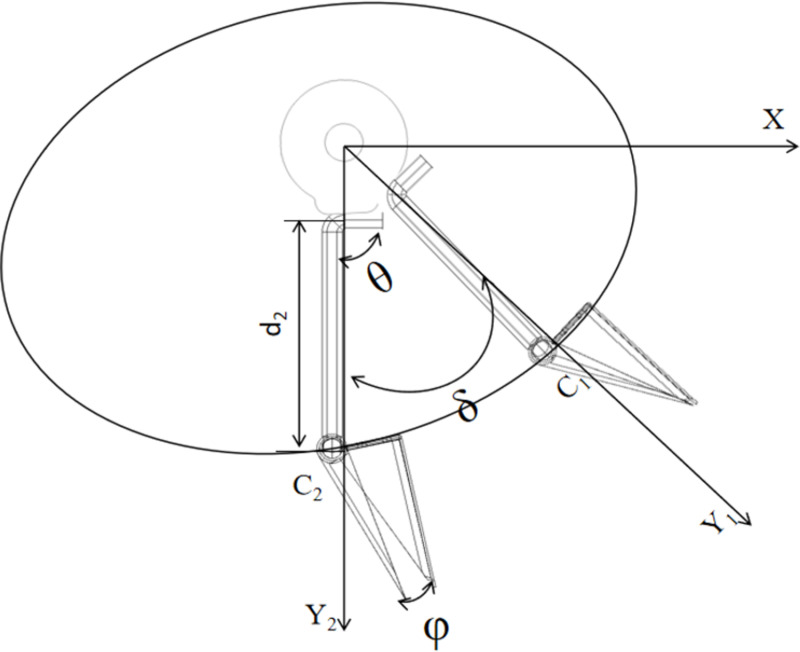
Schematic diagram of the relative motion of the pendulum-lever and the cam.

Due to the special geographical conditions in hilly and mountainous areas, the cavitation device is subjected to large soil resistance during operation, and its velocity and acceleration only change at the beginning and end of the movement during the whole movement process, so it has flexible impact. Therefore, the motion law of cosine acceleration is adopted. Due to the role of the reset spring during operation, only the displacement velocity equation of the moving pendulum- lever rod when it is pushed is analyzed, as shown in Fig 6.

Displacement equation from point C_1_ to point C_2_:


$$\{\begin{array}{l} S_{Xc}=Xc+d_{2c}\cos(\theta -\delta )\\ S_{Yc}=Yc-d_{2c}\sin(\theta -\delta ) \end{array}$$ (9)

Velocity equation from point C_1_ to C_2_:


$$\{\begin{array}{l} V_{Xc}=d_{2c}\sin(\theta -\delta )\phi \\ V_{Yc}=d_{2C}\cos(\theta -\delta )\phi \end{array}$$ (10)

here, δ is The angular displacement turned by the pendulum-lever d_2_.

The angle of the movable pendulum-lever mainly affects the opening degree of the hole formers device, so that it can ensure that the hole-opening time of the sowing work and the seed falling process does not collide with the duckbill. From the above analysis, it can be seen that the movable pendulum-lever angle is a key factor affecting the seeding performance, therefore, it is necessary to determine the optimal movable pendulum-lever angle by combining the displacement and velocity during the movement process as well as the test.

### 2.5. Optimized design of cam group mechanism

**Fig 7:**
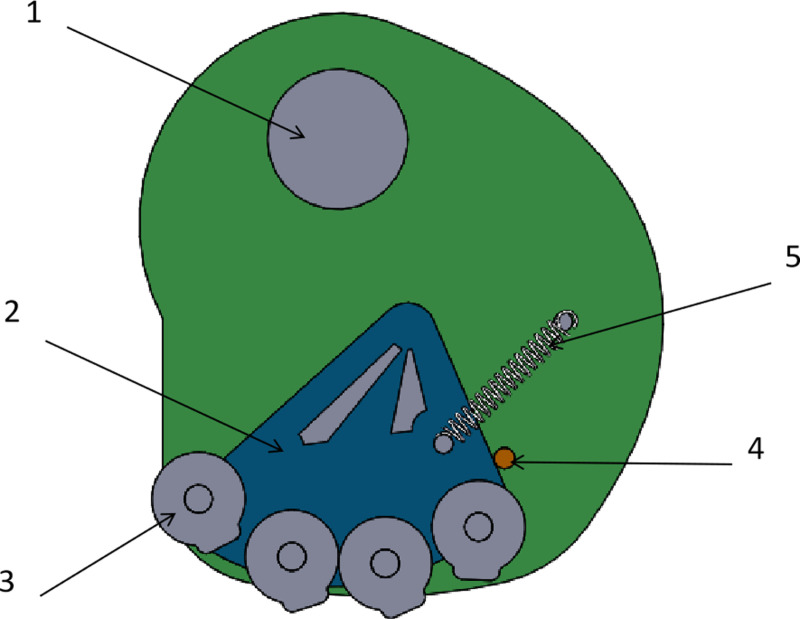
Cam group mechanism. 1. Bearing, 2. Fixing plate, 3. Roller, 4. Limit rod, 5. Return spring.

The cam group mechanism consists of a cam, a fixed plate, a reset spring, a limit lever, and a roller, as shown in Fig 7. During operation, the reset spring keeps the roller set near the limit lever of the fixed plate. The pendulum-lever slides along the outer circle of the roller to ensure that the hole formers device remains open for the seed to fall into the soil. In addition, a limit device on the cam regulates the opening and closing of the hole-former device within the appropriate time frame, facilitating smooth seeding.

#### 2.5.1. Selection of the number of cam rollers.

The cam roller is one of the key components of the hole forming device to realize the function of duckbill opening, and its quantity determines the opening and closing time of the movable duckbill and at the same time determines whether the seeds can fall into the soil smoothly. By precalculation, the seed drop time should fulfill the following conditions.


$$\{\begin{array}{l} \Delta t=\frac{\beta }{\omega }\\ \omega =\frac{v(1-\eta )}{R_{s}-h} \end{array}$$ (11)

here, △t is the time for one seeding of hole seeders; Rs is the radius from the apex of the duckbill to the axis of the hole planter spindle; h is the depth of entry of duckbill into soil; η is the slip rate.

**Fig 8:**
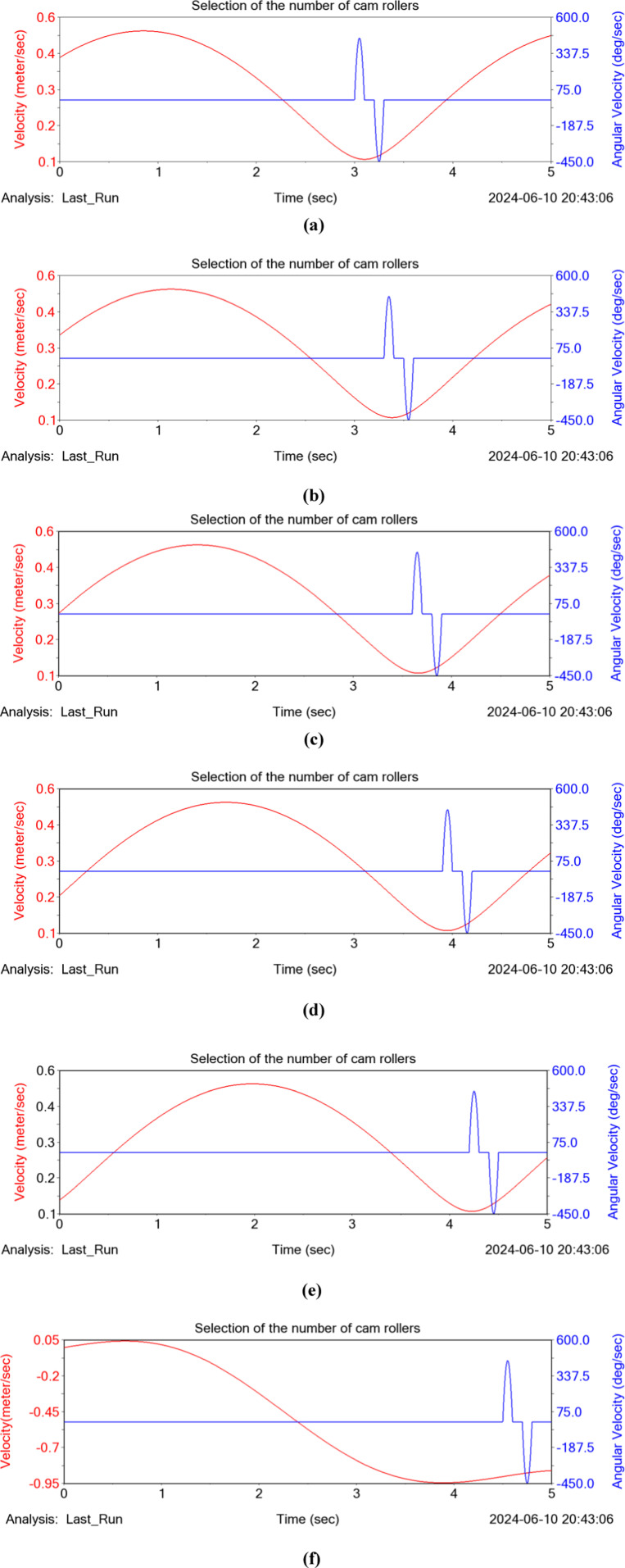
Analysis of forces on pendulum-lever topmost part and cam roller. (a) Cam roller 1, (b) Cam roller 2, (c) Cam roller 3, (d) Cam roller 4, (e) Cam roller 5, (f) Cam roller 6.

In order to optimize the theoretical analysis results, ADAMS simulation is used to obtain the law of the force between the pendulum-lever and the cam roller with time. Save the 3D solid model of the assembly built in SOLIDWORKS as X_T format and import it into ADAMS 2020. In order to facilitate simulation and analysis, the mechanical model is simplified and parts that do not affect the mechanical analysis are deleted, and the relative positions of entities and invariants are integrated through Boolean operations. According to the force applied during the working process of hole formers machine and the relative motion relationship with the cam group roller, add appropriate forces, constraints and drives, and set the simulation time of 5 seconds and the number of working steps of 1000 steps. The force on the top of the pendulum-lever at each moment is shown in Fig 8.

As can be seen from Fig 8, the radial traveling distance of the movable pendulum-lever varies periodically, and the contact force between its tip and the cam roller decreases sequentially from the 1st roller to the 5th roller, and tends to 0 at the 6th roller, which proves that the tip of the pendulum-lever is obviously stressed for the first 5 cam rollers, and that the hole formers device is opened for a total of about 1.2 seconds. The ADAMS simulation software was used to determine the number of rollers in the cam set by using the test factor at the highest rate for the satisfactory rate.

## 3. Simulation test based on DEM-MBD coupling

**Fig 9:**
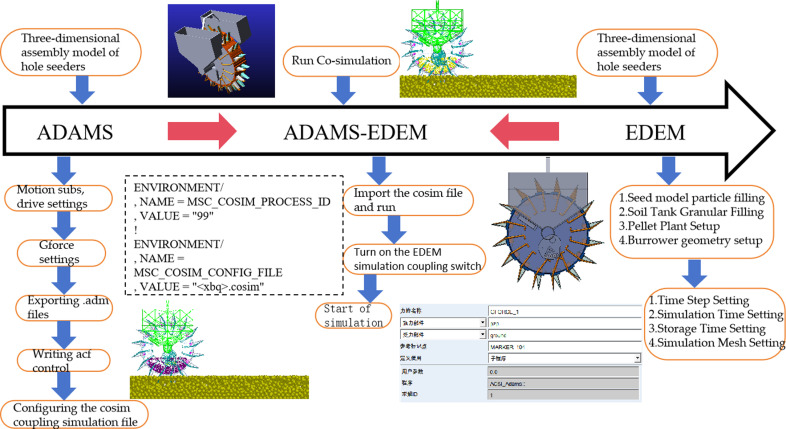
Coupled simulation flow.

Based on the coupled DEM-MBD simulation method for the joint simulation of the hole seeders working device, the simulation process can be roughly divided into three parts: The virtual prototype model is imported in ADAMS, the soil discrete element model is established in EDEM to simulate the real operation field situation in the field, and the joint simulation control file and simulation calculation are written through the ACSI configuration [25–27], and the specific operation steps are shown in Fig 9.

### 3.1. Soil discrete element model

For the Southwest hilly mountainous areas of corn and soybean planting land soil with strong bonding and thick and heavy and other characteristics [28,29] between soil particles using for simulation of viscous wet particles designed for the Hertz-Mindlin with JKR Cohesion Mechanical Contact Model, the mechanical model can be fully considered between the soil particles of the bonding force of the particle movement pattern of the influence. In order to ensure the accuracy and efficiency of the simulation test, the radius of the soil particle model was set to 4 mm, the size of the soil tank was (length ×  width ×  thickness) 2000 mm × 500 mm × 200 mm, and the soil contact parameters were shown in Table 3 [30].

**Table 3:** Soil contact parameters.

Argument	Numerical value
Soil density (kg/m³)	2060
Poisson’s ratio of soil	0.38
Soil shear modulus (pa)	1.05 × 10^10^
Coefficient of static friction between soil particles	0.4
Coefficient of dynamic friction between soil particles	0.22
Recovery coefficient between soil particles	0.2

### 3.2. Discrete meta-modeling of corn and soybean kernels

Seed grain modeling to the southwest region of large-scale use of varieties Yu Dou 11 soybean seeds and Dong dan 1331 corn seeds as test materials, soybean seeds high degree of spherical shape and corn seed shape is irregular, modeling before the randomly selected 1000 corn seed shape classification, divided into flat and spherical two kinds of shape. Particle modeling was carried out for the two shapes of flat and spherical seeds.

**Fig 10:**
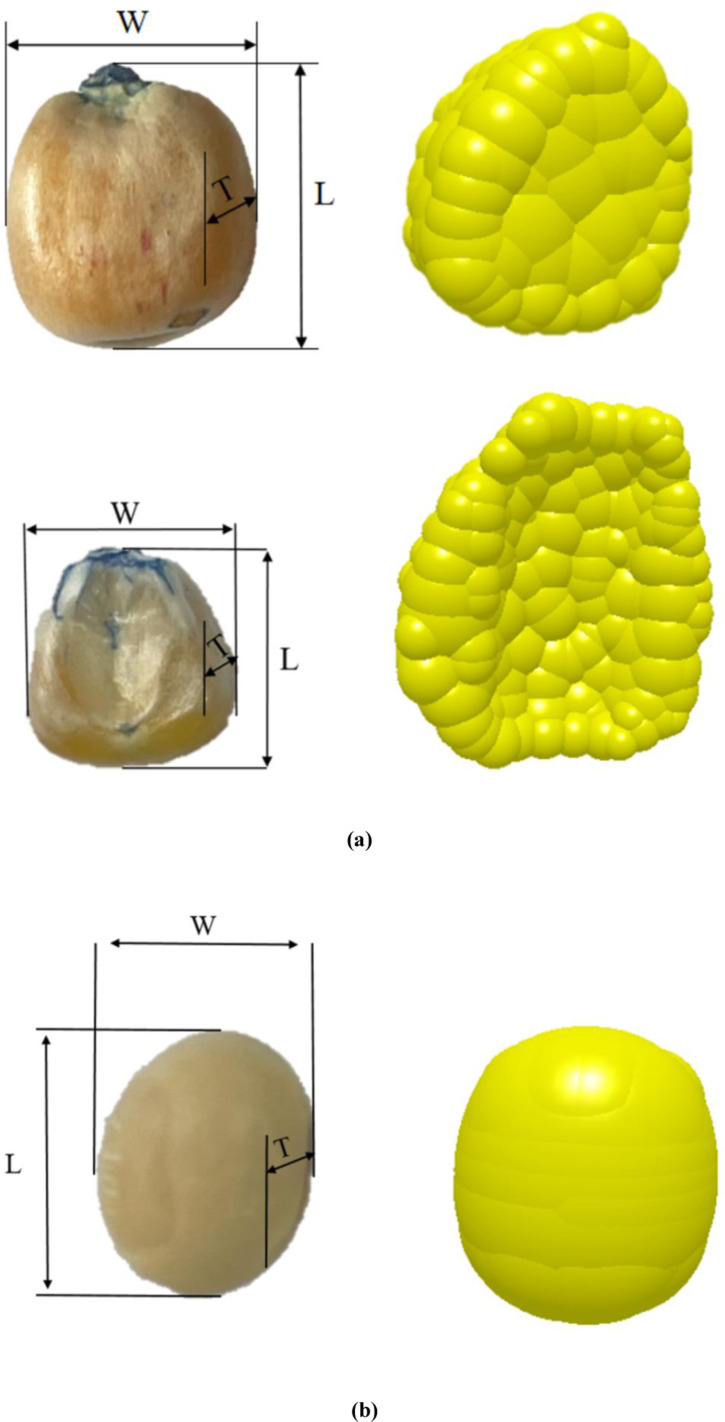
Seed particle model. (a) Corn seed dimensions, (b) Soybean seed dimensions.

Hertz-Mindlin (no slip) contact model was chosen for the particle contact model. Spherical seeds were selected to be filled with a smoothing value of 3, and the smallest ball with a radius of 1 mm; flattened seeds, due to the thickness from the bottom to the upper part of which was gradually smaller, were selected to be filled with a smoothing value of 5, and the smallest ball with a radius of 1.5 mm. Soybean seeds have a single, ellipsoidal form factor and are modeled as particles based on the soybean triaxial dimensions above. Seed particle modeling is shown in Fig 10.

The contact models for the pellet-to-pellet and pellet-to-Hole seeder models were all Hertz-Mindlin (no-slip) no-slip contact models. The parts of the Hole seeder that come into contact with the seed are the Hole seeder housing, the Hole formers device, the seed box, and the seed pickup block, where the Hole seeder housing, the seed box, and the seed pickup block are nylon and the Hole formers device is stainless steel. After parametric calibration, the intrinsic parameters of seed particles, nylon and steel and the contact parameters between them are shown in Table 4 [31].

**Table 4:** Discrete element simulation parameters.

Argument	Soybean	Corn	Nylon	Stainless steel
Density (kg/m³)	1232	1162	1250	7861
Poisson’s ratio	0.23	0.38	0.34	0.30
Shear modulus (pa)	6.04 × 10^6^	2.08 × 10^8^	3.0 × 10^9^	7.19 × 10^10^
Static friction coefficient with soybean	0.39		0.51	0.50
Coefficient of dynamic friction with soybean	0.17		0.03	0.01
Collision recovery coefficient with soybean	0.30		0.31	0.53
Coefficient of static friction with corn		0.50	0.38	0.37
Coefficient of dynamic friction with corn		0.01	0.09	0.01
Collision recovery coefficient with corn		0.55	0.45	0.60

**Fig 11:**
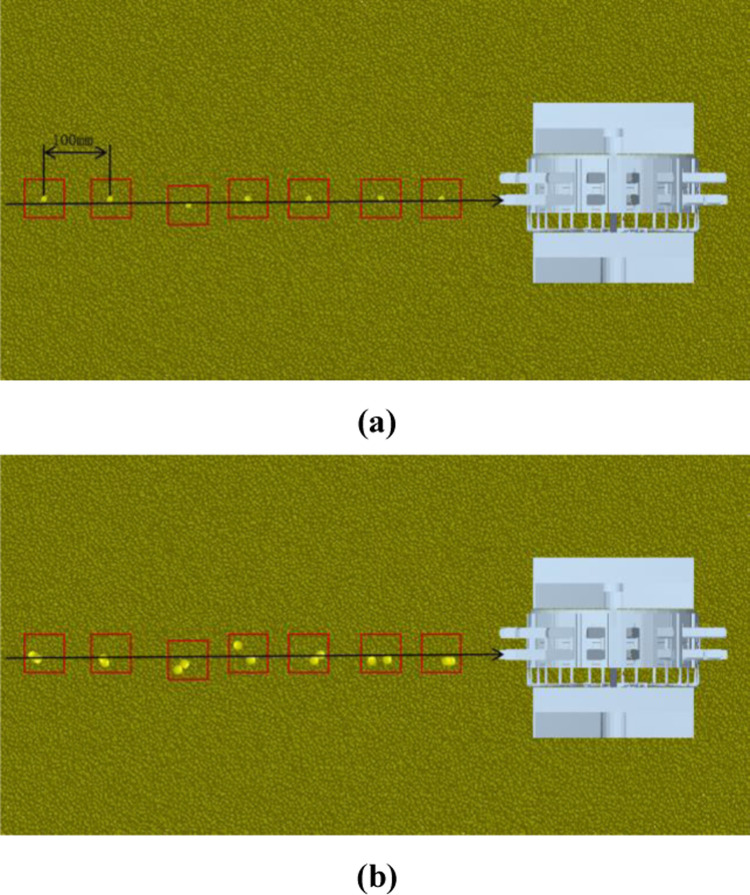
Schematic diagram of seeds falling on soil particles.

The EDEM software was used to establish the seed grain-soil discrete element model, the seed grain was filled with particles, the contact model was Hertz-Mindlin with bonding, and the simulation of the hole seeders planter for open-hole seeding was shown in Fig 11.

### 3.3. Single factor experiment and result analysis

Based on the previous theoretical analysis and presetting pretests, it is known that the main factors affecting the seeding performance of the hole seeders are the rotational speed of hole seeder, the angle of the movable pendulum-lever and the number of rollers of the cam group. Successively carry out the single-factor test and quadratic regression orthogonal rotary combination test of seed arrangement with the rotational speed of hole seeders plant rotor shaft, the angle of movable pendulum-lever and the number of rollers of cam group, and explore the influence law of each factor on the number of qualified number, the number of reseeded and the number of omission of seeding. The intermediate levels of the one-factor test were set as follows: The speed of the hole seeder is 60 r/min, the angle of the movable pendulum-lever is 90°, the number of rollers of cam set is 3, and the factors and levels are shown in Table 5.

**Table 5:** Experimental factors and levels for seeding simulation with hole seeders.

Level	Experimental factor		
	Rotational speed of hole seeder (r/min)	The movable pendulum-lever angle (°)	Number of rollers of cam set (pieces)
	A	B	C
1	40	70	1
2	50	80	2
3	60	90	3
4	70	100	4
5	80	110	5

#### 3.3.1. Influence of rotational speed of hole seeder on various performance indicators.

**Fig 12:**
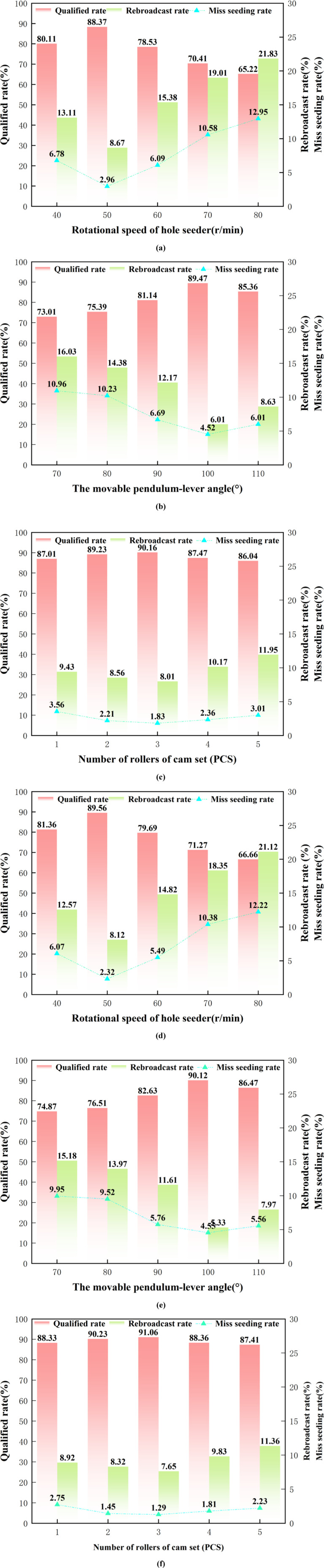
Results of one-factor simulation tests for corn and soybean. (a) and (d) is the Influence of rotation speed of the hole seeder on each index, (b) and (e) is the influence of swinging rod Angle on each index, (c) and (f) is the influence of roller number of cam set on each index. (Please refer to the attachment for relevant S3 Fig. 12 data).

The angle of the movable pendulum-lever is 90°, the number of rollers in the cam group is 3, analyze the impact on seed discharge performance when the rotational speed of the hole seeder is 40, 50, 60, 70, 80 r/min respectively, and the test results are shown in Fig 12.

From Fig 12a and 12d, when the rotary speed of the hole seeders rotation speed was increased from 40 r/min to 80 r/min, the qualified rate of corn and soybean decreased with the increase of the rotary speed of the hole seeders, and the qualified rate reached the lowest value of 65.22% and 66.66%, respectively, with the decrease of 14.89% and 14.7% when the rotary speed of the hole seeders rotation speed was 80 r/min. As the rotational speed of the hole seeders rotation speed increased from 50 r/min to 80 r/min, the qualified rate began to decrease with the increase of the rotational speed of the hole seeders. This indicates that increasing the rotational speed of the hole seeders rotation speed reduces the qualified rate. The rebroadcast rate showed a decreasing and then increasing trend with the increase of the rotational speed of hole seeders, when the rotational speed of hole seeders rotation speed was increased from 50 r/min to 80 r/min, the rebroadcast rate increased from 8.67% and 8.12% to 21.83% and 21.12%, respectively, which was an increase of 13.16% and 13%.

#### 3.3.2. Influence of movable pendulum-lever on various performance indicators.

The rotational speed of the hole seeder is 60 r/min, the number of rollers in the cam group is 3, analyze the impact on seed discharge performance when The angle of the movable pendulum-lever is 70, 80, 90, 100, 110° respectively.

As can be seen from Fig 12b and 12e, as the angle of the movable pendulum-lever increased from 70° to 110°, the miss seeding rate of corn and soybean showed a trend of decreasing and then increasing, and the miss seeding rate reached the lowest value of 4.52% and 4.55%, respectively, at the angle of the movable pendulum-lever of 100°, with a decrease of 5.4% and 6.44%, respectively, compared with that at the angle of the movable pendulum-lever of 70°. As the movable pendulum-lever angle increases from 100° to 110°, the miss seeding rate begins to increase. This indicates that the movable pendulum-lever angle at 100° is the inflection point of the miss seeding rate. Qualified rate with the increase of the angle of the movable pendulum-lever showed a decreasing and then rising trend, in the movable pendulum-lever angle of 100° when the qualified rate reached the maximum value of 89.47% and 90.12%.

#### 3.3.3. Influence of number of rollers cam set on various performance indicators.

The rotational speed of the hole seeder is 60 r/min, The angle of the movable pendulum-lever is 90°, analyze the impact on seed discharge performance when the number of rollers in the cam group is 1, 2, 3, 4, 5 respectively.

As can be seen in Fig 12c and 12f, as the number of rollers in the cam set increased from 1 to 3, the leakage rate of corn and soybean decreased from 2.75% and 3.56% to 1.29% and 1.83%, respectively. The miss seeding rate decreases with the increase of the number of rollers in the cam set with a negative correlation, which is consistent with the theoretical analysis. Replay rate with the increase in the number of rollers in the cam group shows a decreasing and then increasing trend, and reaches its lowest value when the number of rollers in the cam group is 3, which is 7.65% and 8.01%, respectively. The qualified rate peaked at 91.06% and 90.16% when the number of rollers in the cam set was 3, respectively.

### 3.4. Quadratic regression orthogonal rotation combination test

#### 3.4.1. Test methods and indicators.

According to the results of the one-way test, when the rotational speed of the hole seeder is 40–60 r/min, the angle of the movable pendulum-lever is 90°–110°, and the number of rollers in the cam group is 2–4, the seeding qualified rate is higher, and the reseeding rate and leakage rate are lower. Based on the results of the one-way test, the rotational speed of the hole seeders rotor, the angle of the movable pendulum-lever, and the number of rollers of the cam group were taken as the design variables, and the qualified rate, the rebroadcast rate, and the miss seeding rate were taken as the objective functions, respectively, and the coding of each design variable is shown in Table 6.

**Table 6:** Design variable coding.

Encoding	X_1_(r/min)	X_2_(°)	X_3_(mm)
−1	40	90	2
0	50	100	3
1	60	110	4

The quadratic regression orthogonal rotating combination test was designed according to Box-Behnken response surface method, and the test program and results are shown in Table 7.

**Table 7:** Simulation test program and results of seed discharging by hole seeder.

Test number	Corn						Soya bean					
	Factor level			Test index			Factor level			Test index		
	X_1_	X_2_	X_3_	Q/%	R/%	L/%	X_1_	X_2_	X_3_	Q/%	R/%	L/%
1	−1	−1	0	83.25	4.62	12.13	−1	−1	0	84.17	3.57	12.26
2	1	−1	0	75.69	4.53	19.78	1	−1	0	76.38	3.44	20.18
3	−1	1	0	88.58	10.09	1.23	−1	1	0	89.17	9.12	1.71
4	1	1	0	79.83	9.88	10.29	1	1	0	80.63	8.96	10.41
5	−1	0	−1	91.37	5.69	2.94	−1	0	−1	92.33	4.39	3.28
6	1	0	−1	85.69	5.47	8.84	1	0	−1	86.79	4.21	9.00
7	−1	0	1	78.28	7.78	13.94	−1	0	1	79.41	6.88	13.71
8	1	0	1	69.41	7.21	23.38	1	0	1	70.33	6.36	23.31
9	0	−1	−1	90.26	3.47	6.27	0	−1	−1	91.12	2.35	6.53
10	0	1	−1	87.61	8.24	4.15	0	1	−1	88.67	7.62	3.71
11	0	−1	1	68.47	4.58	26.95	0	−1	1	69.32	3.55	27.13
12	0	1	1	86.24	11.28	2.48	0	1	1	87.42	10.03	2.55
13	0	0	0	92.33	4.56	3.11	0	0	0	93.35	3.49	3.16
14	0	0	0	93.45	4.56	1.99	0	0	0	94.65	3.49	1.86
15	0	0	0	93.45	4.57	1.98	0	0	0	94.65	3.48	1.87
16	0	0	0	94.27	4.26	1.47	0	0	0	95.11	3.31	1.58
17	0	0	0	92.41	4.22	3.37	0	0	0	93.47	3.22	3.31

#### 3.4.2. Analysis of test results.

A multiple regression was fitted to the test results using Design-Expert 13 software and the significance test results are shown in Table 8.

**Table 8:** Analysis of variance for response surface regression models.

Sources of Variance	Q				R					L			
	Square of Sum	Degree of Freedom	F	P	Square of Sum	Degree of Freedom	F		P	Square of Sum	Degree of Freedom	F	P
Corn													
Model	1106.07	9	66.32	0.0001	97.07	9	275.03		0.0001	1062.28	9	65.42	0.0001
X_1_	119.74	1	64.61	0.0001	0.1225	1	3.12		0.0205	127.52	1	70.68	0.0001
X_2_	77.50	1	41.82	0.0043	65.09	1	1659.93		0.0001	284.65	1	157.77	0.0132
X_3_	343.61	1	185.42	0.0335	8.51	1	216.95		0.0031	243.98	1	135.23	0.0302
X_1_X_2_	0.1406	1	0.0759	0.7909	0.0002	1	0.0057		0.9417	0.1521	1	0.0843	0.7800
X_1_X_3_	3.13	1	1.69	0.2347	0.0289	1	0.7370		0.4191	3.76	1	2.09	0.1919
X_2_X_3_	105.58	1	56.97	0.0001	0.3660	1	9.33		0.0184	118.37	1	65.61	0.0034
X_1_^2^	194.01	1	104.69	0.0001	6.30	1	160.73		0.0688	130.37	1	72.26	0.0001
X_2_^2^	99.88	1	53.90	0.0034	11.48	1	292.67		0.0001	43.64	1	24.19	0.0017
X_3_^2^	115.74	1	62.46	0.0466	2.96	1	75.49		0.0371	81.68	1	45.27	0.0003
Residual error	12.97	7			0.2745	7				12.63	7		
Lack-of-fit	10.49	3	5.65	0.5102	0.2114	3	4.47		0.0911	9.99	3	5.04	0.0760
Error	2.48	4			0.0631	4				2.64	4		
Sum	1119.04	16			97.34	16				1074.91	16		
Soya bean													
Model	1091.49	9	71.66	0.0001	93.80	9		328.72	0.0001	1055.70	9	63.05	0.0001
X_1_	119.04	1	70.34	0.0001	0.1485	1		4.368	0.0672	128.40	1	69.02	0.0001
X_2_	75.58	1	44.66	0.0003	62.11	1		1958.77	0.0001	275.89	1	148.30	0.0001
X_3_	344.93	1	203.80	0.0001	7.96	1		251.05	0.0001	248.09	1	133.35	0.0001
X_1_X_2_	0.3540	1	0.2092	0.6613	0.0036	1		0.1135	0.7460	0.4970	1	0.2672	0.6212
X_1_X_3_	2.54	1	1.50	0.2598	0.0306	1		0.9659	0.3584	3.13	1	1.68	0.2355
X_2_X_3_	104.24	1	61.59	0.0001	0.9312	1		29.37	0.0010	124.88	1	67.13	0.0001
X_1_^2^	186.26	1	110.05	0.0001	6.53	1		206.00	0.0001	122.46	1	65.82	0.0001
X_2_^2^	92.75	1	54.80	0.0001	10.79	1		340.17	0.0001	39.96	1	21.48	0.0024
X_3_^2^	120.22	1	71.03	0.0001	3.10	1		97.76	0.0001	85.19	1	45.79	0.0003
Residual error	11.85	7				7				13.02	7		
Lack-of-fit	9.20	3	4.63	0.0864		3		1.01	0.4758	10.37	3	5.21	0.0723
Error	2.65	4				4				2.65	4		
Sum	1103.34	16				16				1068.73	16		

#### 3.4.3. The effect of the interaction of factors on the qualified rate.

**Fig 13:**
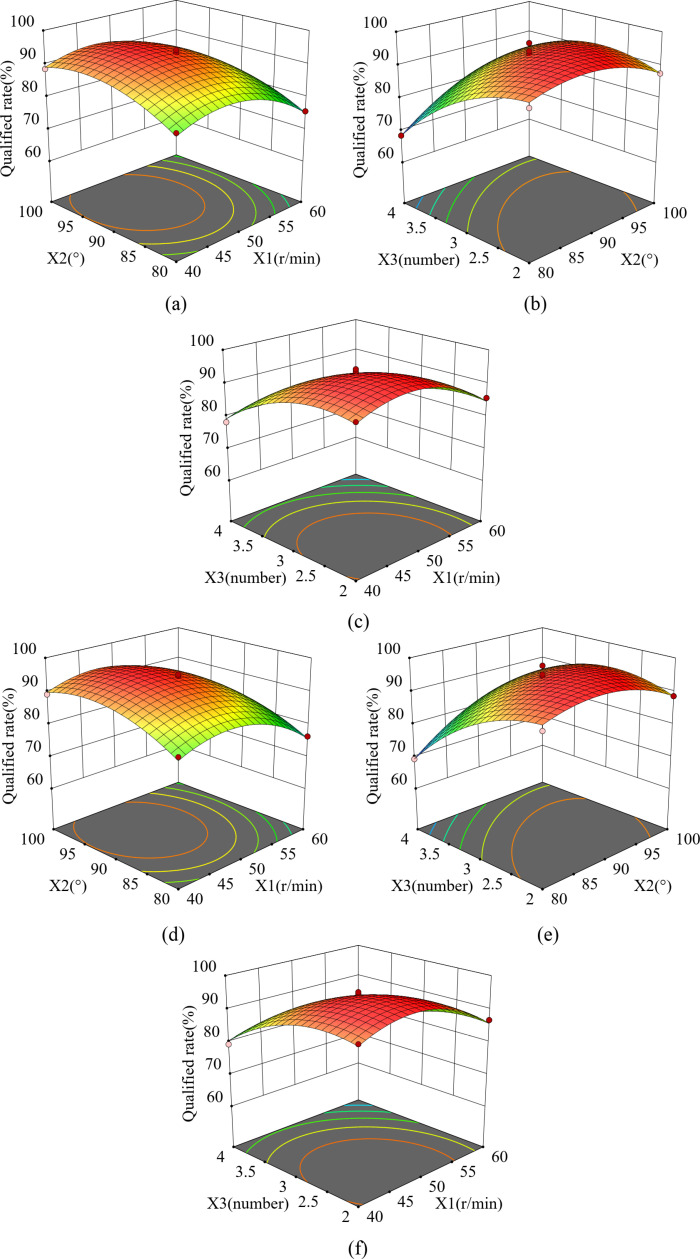
Effect of interactions on qualified rate. (a) and (d) Interaction between X_1_X_2_, (b) and (e) Interaction between X_1_X_3_, (c) and (f) Interaction between X_2_X_3_.


The effects of the interaction among factors on the qualified rate of corn and soybean sowing are shown in Fig 13a–13c and 13d–13f below, respectively. The P-values of X_1_, X_2_, X_2_X_3_, X_1_^2^, and X_2_^2^ are all less than 0.01, which indicates that the effects on the qualified rate are extremely significant; The P-values of X_3_, X_3_^2^ are greater than 0.01 and less than 0.05, which is significant for the qualified rate; The rest of the items have no significant effects on the qualified rate; the factors affecting the qualified rate are, in the order of priority, the speed of hole seeders rotor, the angle of movable pendulum-lever, and the number of rollers of the cam group. The influence of the remaining items on the qualification rate is not significant, and the factors affecting the qualification rate in the order of priority are the rotational speed of the rotor shaft of the hole seeders, the angle of the movable pendulum-lever and the number of rollers of the cam group. The regression equation for the qualified rate Q is:

$$\begin{array}{l} Q_{\text{Corn}}=93.18-3.86x_{1}+3.07x_{2}-6.57x_{3}\\ -0.7975x_{2}x_{3}-6.65x_{1}^{2}-4.69x_{2}^{2}-5.34x_{3}^{2} \end{array}$$ (12)

$$\begin{array}{l} Q_{\text{Soya bean}}=94.25-3.87x_{1}+3.11x_{2}-6.55x_{3}\\ -0.8850X_{2}X_{3}-4.87x_{2}^{2}-5.24x_{3}^{2} \end{array}$$ (13)

#### 3.4.4. The effect of the interaction of factors on the rebroadcast rate.

**Fig 14:**
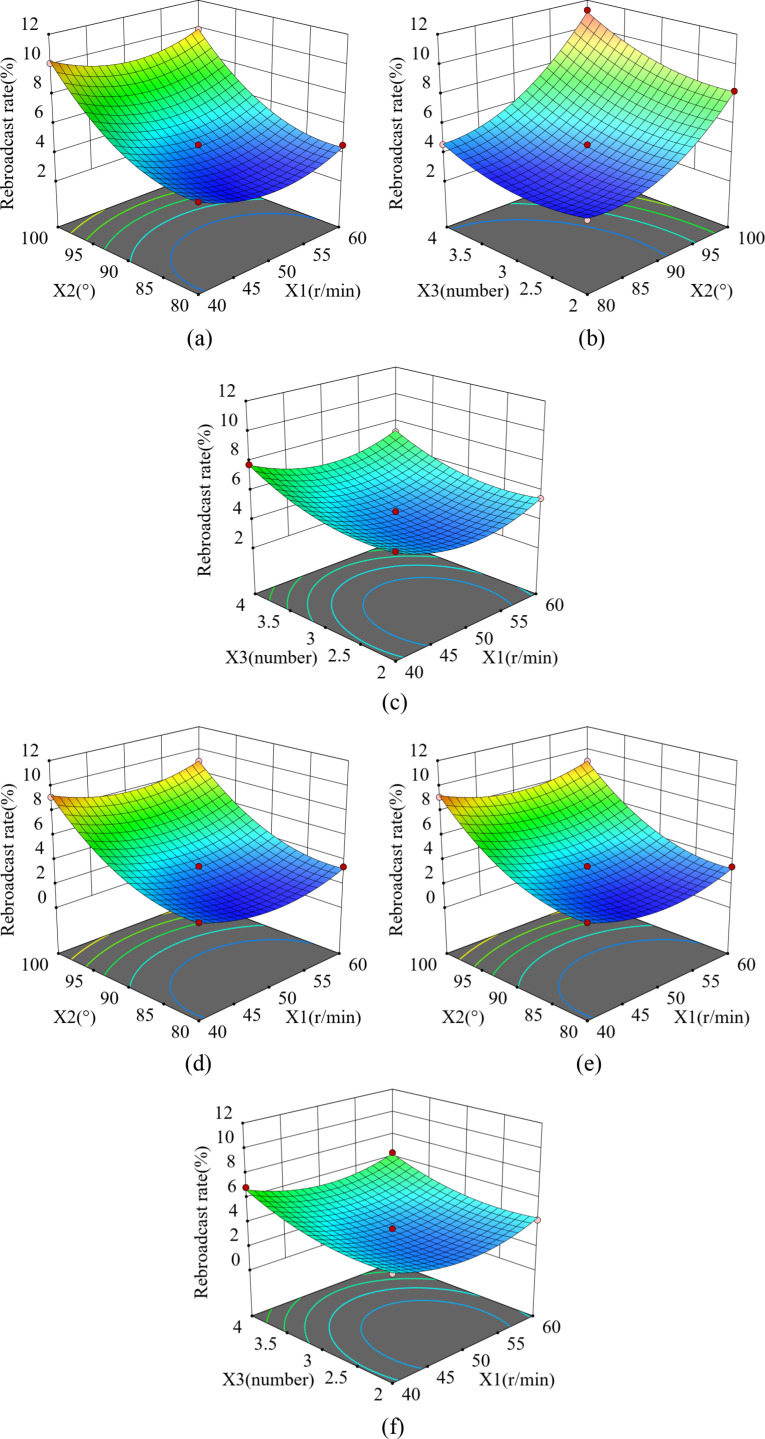
Effect of interaction on rebroadcast rate. (a) and (d) Interaction between X_1_X_2_, (b) and (e) Interaction between X_1_X_3_, (c) and (f) Interaction between X_2_X_3_.


The effects of the interactions among the factors on the seeding qualified rate of corn and soybean are shown in Fig 14a–14c and 14d–14f below, respectively. The P-values of X_2_, X_3_, and X_2_^2^ are all less than 0.01, which indicates that the effects on replay rate are extremely significant; the P-values of X_1_, X_2_X_3_, and X_3_^2^ are greater than 0.01 and less than 0.05, which indicate that the effects on rebroadcast rate are significant; the effects of the rest of the items on rebroadcast rate are not significant. The factors affecting rebroadcast rate were, in order of predominance, the angle of the movable pendulum-lever, the number of rollers in the cam set and the rotational speed of the hole seeders spindle. The regression equation for rebroadcast rate R is:

$$\begin{array}{l} R_{\text{Corn}}=4.43+2.79x_{2}+0.9975x_{3}\\ +0.4825x_{2}x_{3}+1.25x_{1}^{2}+1.60x_{2}^{2}+0.8580x_{3}^{2} \end{array}$$ (14)

$$\begin{array}{l} R_{\text{Soya bean}}=3.40-0.1237x_{1}+2.85x_{2}+1.03x_{3}\\ +0.3025X_{2}X_{3}+1.65x_{2}^{2}+0.8385x_{3}^{2} \end{array}$$ (15)

#### 3.4.5. The effect of the interaction of factors on miss seeding rate.

**Fig 15:**
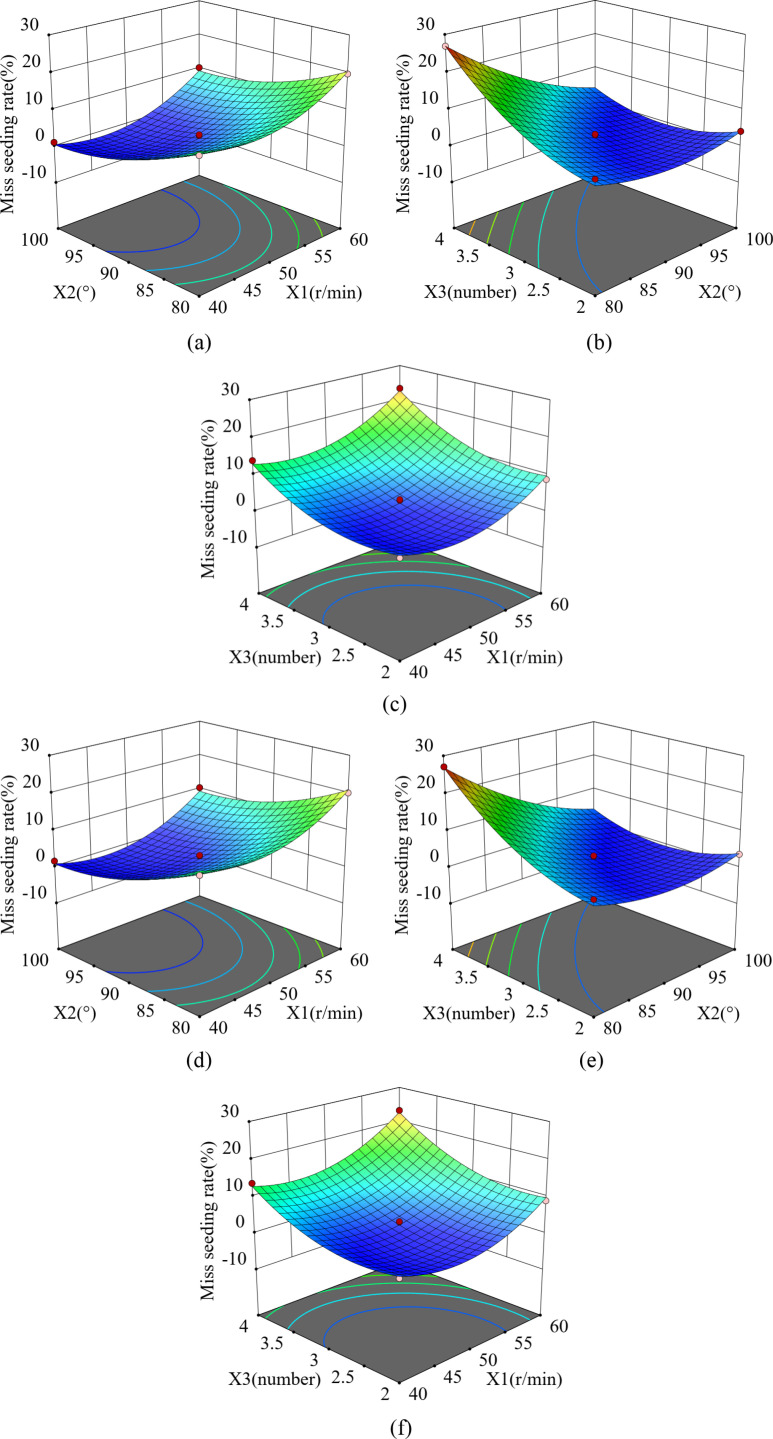
Effect of interaction on miss seeding rate. (a) and (d) Interaction between X_1_X_2_, (b) and (e) Interaction between X_1_X_3_, (c) and (f) Interaction between X_2_X_3_.


The effects of the interaction among factors on the seeding qualified rate of corn and soybean are shown in Fig 15a–15c and 15d–15f below, respectively. The P-values of X_1_, X_2_X_3_, X_1_^2^, X_2_^2^, and X_3_^2^ are all less than 0.01, which indicates that the effects on the miss seeding rate are extremely significant; The P-values of X_2_ and X_3_ are larger than 0.01 and smaller than 0.05, which is significant to the miss seeding rate; and the rest of the items have no significant effect on the miss seeding rate. The factors affecting the miss seeding rate were, in order of priority, the rotational speed of the rotor shaft of the hole seeders, the angle of the movable pendulum-lever and the number of rollers of the cam group. The regression equation of miss seeding rate L is:

$$\begin{array}{l} L_{\text{Corn}}=2.38+4.01x_{1}-5.87x_{2}+5.57x_{3}-5.59X_{2}X_{3}\\ +5.39x_{1}^{2}+3.08x_{2}^{2}+4.50x_{3}^{2} \end{array}$$ (16)

$$\begin{array}{l} L_{\text{Soya bean}}=2.36+2.36x_{1}-5.86x_{2}+5.52x_{3}-5.44X_{2}X_{3}\\ +5.56x_{1}^{2}+3.22x_{2}^{2}+4.40x_{3}^{2} \end{array}$$ (17)

### 3.5. Optimization of test result objectives

In order to seek the optimal combination of factors under the level constraints, the qualified rate、rebroadcast rate and miss seeding rate are still taken as the evaluation indexes, the mathematical model is established by combining the boundary conditions of the factors, and the regression model of the evaluation indexes is solved by multi-objective, and the optimization objective function and constraint conditions are as follows:


$$\{\begin{array}{l} \max Y_{1}(X_{1},X_{2},X_{3})\\ \min Y_{2}(X_{1},X_{2},X_{3})\\ \min Y_{3}(X_{1},X_{2},X_{3})\\ 40\leq X_{1}\leq 60\\ 90\leq X_{2}\leq 110\\ 2\leq X_{3}\leq 4 \end{array}$$ (18)

With the highest qualified rate, rebroadcast rate and the lowest miss seeding rate as the optimization objective for multi-objective optimization, after solving for different hole number requirements of corn and soybean hole seeders rotation speed of 47.43 r/min and 48.09 r/min, respectively, the movable pendulum-lever angle of 100.23 ° and 101.70 °, respectively, the number of rollers of the cam group of 2.81 and 2.95 when the optimal performance, respectively, 95.19 and 96.07% qualified rate, 3.58% and 2.35%, miss seeding rate of 1.23% and 1.58%. The qualified rates were 95.19 and 96.07%, the rebroadcast rate were 3.58% and 2.35%, and the miss seeding rate were 1.23% and 1.58%, respectively.

### 3.6. Field trial

**Fig 16:**
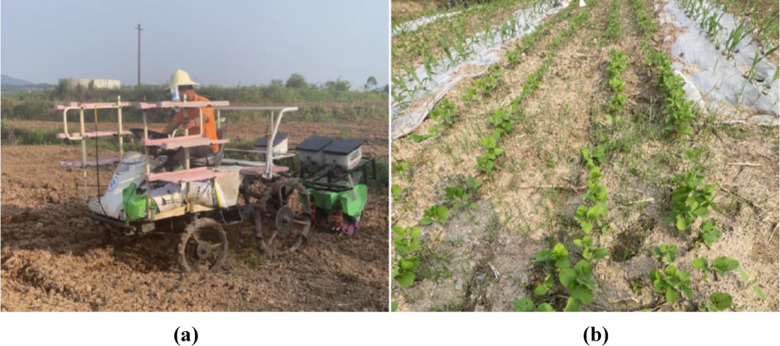
Field trial. (a) Test site, (b) Emergence condition. (Please refer to the attachment for relevant data S1 Fig. 16 original photo).

On April 28, 2024, a field operation performance test of a mountain soybean corn ribbon composite planter was carried out at the standardized farmland planting test base in Jiaopengjing Village, Yongchuan District, Chongqing Municipality, as shown in Fig 16. The test site is loamy soil, good land preparation, in line with the agronomic requirements of corn and soybean planting, the test soybean varieties for Yudou 11 and corn varieties for Dongdan 1331, straightedge (500 mm), tape measure (5 m), with reference to the GB/T 6973-2005 “single grain (precision) planter test methods” to determine the qualified indicators in the field test, the leakage sowing index and re-sowing index. Due to the hilly and mountainous areas of land fragmentation of small, undulating terrain to take advantage of rice transplanter head turning radius is small, adaptable characteristics, supporting power for the Yanmar 2ZGZ-6 (VP6E) type rice transplanter head for field trials.

### 3.7. Test result

**Fig 17:**
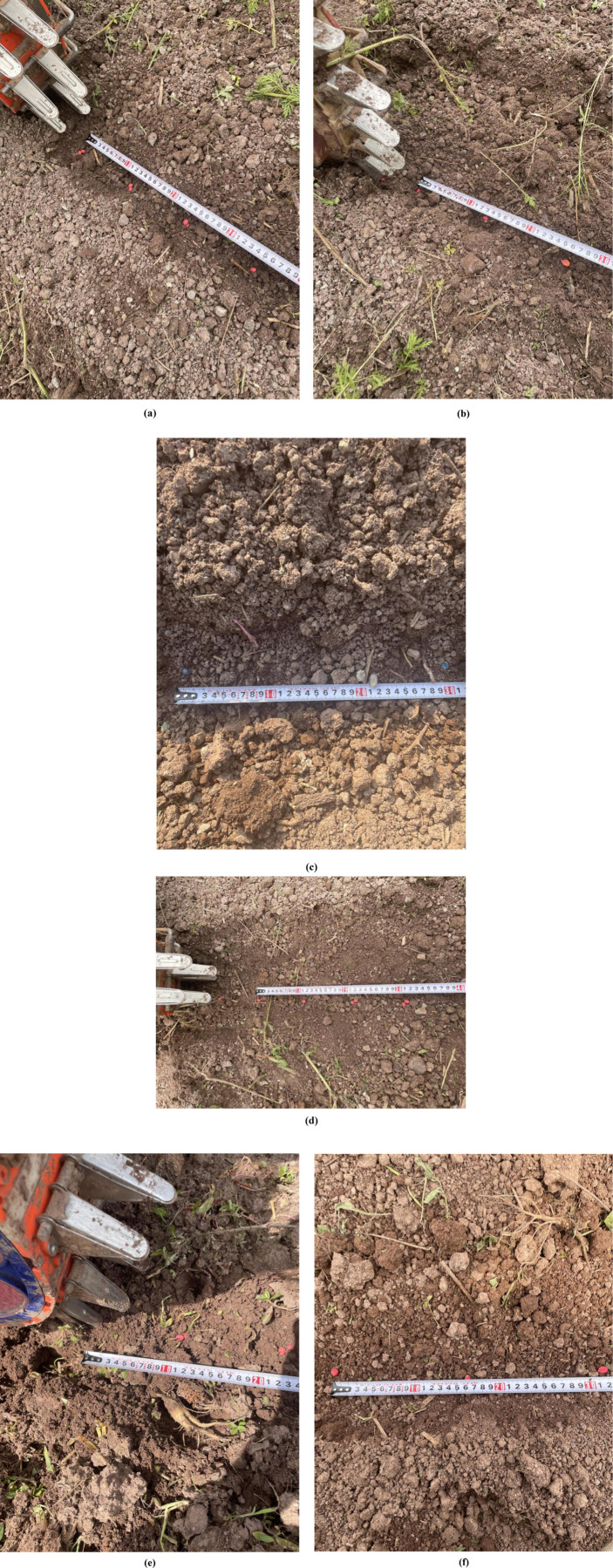
Test results before and after optimization. (a–c) is the Optimize the number of qualified pre-hole particles, (d–f) is the Qualified number of holes after optimization. (Please refer to the attachment for relevant data S2 Fig. 17 original photo).

In order to reduce the test error, the length of the test area is 20 m, the sowing start and end sections are each reduced by 5 m, the middle 10 m is the data collection area, randomly measure the data within 30 m of the operating area, each group of tests continuously record 200 sowing qualified number, rebroadcast number, miss seeding number, repeat the test 3 times. The test results before and after optimization are shown in Fig 17.

In Fig 17, the pre-optimization hole number pass rate was 130 mm for soybeans and 300 mm for corn according to agronomic requirements, while the post-optimization hole number pass rate was 105 mm for soybeans and 150 mm for corn, which indicates that the optimized hole planter usually has a higher and more stable hole formation effect than the original hole planter. In addition, the optimized simulation results did not differ much from the predicted regression model results, which means that the regression model used for parameter optimization was accurate.

Through the constraints and optimization solving function of Design-Expert 13, the optimal rotation speed of the hole seeders for sowing corn and soybeans with mountain pendulum-lever cam-type hole seeders are determined to be 47.43 r/min and 48.09 r/min, the movable pendulum-lever angles are 100.23° and 101.70°, and the number of rollers in the cam group is 3. The actual roller number of cam group is an integer, so the roller number prediction value is 2 to 2.5, the roller number is 2, the roller number prediction value is 2.5 to 3.5, the roller number is 3, and the cam group actual roller number is 3. The actual number of rollers in the cam group is an integer, so when the predicted value of the number of rollers is from 2 to 2.5, the number of rollers is taken as 2, when the predicted value of the number of rollers is from 2.5 to 3.5, the number of rollers is taken as 3, and when the predicted value of the number of rollers is from 3.5 to 4, the number of rollers is taken as 4. According to the optimization results, field trials were carried out to verify the results, and under this parameter, we obtained the corn and soybean qualified rate of 94.79% and 95.94%, respectively, the rebroadcast rate of 2.41% and 2.71%, respectively, and the miss seeding rate of 2.80% and 1.35%, respectively. The results are shown in Table 9.

**Table 9:** Field test results.

	Serial number	Total number of holes	Corn			Soya bean		
			Qualified rate of hole number Q/%	Rebroadcast rate R/%	Miss seeding rate L/%	Qualified rate of hole number Q/%	Rebroadcast rate R/%	Miss seeding rate L/%
Pre-optimization hole seeder	1	200	90.21	5.98	3.81	92.45	3.67	3.88
	2	200	89.88	6.22	3.90	91.16	4.52	4.32
	3	200	89.93	6.01	4.06	91.23	4.34	4.43
	Mean value		90.01	6.07	3.92	91.61	4.18	4.21
Post-optimization hole seeder	1	200	95.33	2.36	2.31	96.21	2.51	1.28
	2	200	94.15	2.01	3.84	95.54	3.17	1.29
	3	200	94.89	2.86	2.25	96.07	2.46	1.47
	Mean value		94.79	2.41	2.80	95.94	2.71	1.35

## 4. Discussion

In this paper, the seeding performance of the pendulum-lever cam-type hole seeder under the condition of clay-heavy soil in hilly and mountainous areas is investigated, and the results are satisfactory. Compared with the existing mechanical hole seeder, the hole seeder studied in this paper mainly highlights two points of optimization design: on the one hand, the active pendulum-lever angle is calculated by the cosine acceleration law of motion in order to accurately adjust the hole seeders opener opening degree to ensure its seeding efficiency under the appropriate angle. On the other hand, the cyclic variation diagram of the active pendulum-lever angle obtained by using ADAMS kinematic simulation test shows that the configuration of multiple cam group rollers makes the hole seeders opening stable in the position of the hole seeders area under the viscous soil, which reduces the phenomena of rebroadcast and miss seeding.

Although the optimized hole seeder can basically meet the requirements of agronomic seeding from an overall point of view, it is obvious from the tests on the actual planter that the effect still needs to be improved. Since there is a machine vibration problem inside the planter during the actual seeding process, the effect of vibration on the hole-seeder effect will be analyzed and optimized in the later stages of this study. An in-depth study of the reliability and durability of the processed materials used in the hole-forming device will be carried out.

## 5. Conclusions

Aiming at the problems of poor hole formation and high miss seeding rate of the existing pendulum-lever cam hole seeders under the operating environment of sticky and heavy soil in hilly and mountainous areas, this study innovatively proposes the effects of different active pendulum-lever angles and the number of rollers of the cam group on the hole formation mechanism. The force changes of the interaction between the pendulum-lever and the rollers were analyzed during the hole-forming and seeding process, and the related structural and working parameters were determined, which included the theoretical analysis of the parameters of the key components and the performance test in the field. The main conclusions are as follows:

(1) Compared with the traditional pendulum-lever cam type hole seeders, the opening and closing of the fixed and movable duckbill have good hole formation and hole spacing uniformity for clayey soil in hilly and mountainous areas, and good adaptability to two types of seeds of different sizes, corn and soybean.
(2) According to the quadratic regression orthogonal rotation combination simulation test and surface response analysis method, when the rotational speed of the hole seeders is 47.43 r/min and 48.09 r/min, the active pendulum-lever angle is 100.23° and 101.70°, and the number of rollers in the cam group is 3, the qualified rate is 94.79% and 95.94%, the rebroadcast rate is 2.41% and 2.71%, and miss seeding rate is 2.80% and 1.35%.
(3) The results of the field test showed that the optimized pendulum-lever cam-type hole seeders increased the qualified rate by 4.78% and 4.33%, the rebroadcast rate decreased by 3.66% and 1.47%, and miss seeding rate decreased by 1.12% and 2.86%, respectively, compared with the pre-optimization one, which verified that the optimized pendulum-lever cam-type corn and soybean hole seeders had higher sowing reliability.

In summary, although the key parameters of the hole-forming device have been studied in this paper, and the optimal set of hole seeders speed, active pendulum-lever angle and cam roller number have been obtained, which can provide a reference for the corn and soybean banded composite planter in hilly mountainous areas. However, if the research results are applied to engineering practice, other variables need to be considered to further improve and optimize the design of the hole-former, including in-depth studies on seed varieties, soil conditions and operating environments, so as to ensure seeding performance under a range of field conditions.

## Supporting information

S1 Fig 16 original photo. (DOCX)

S2 Fig 17 original photo. (DOCX)

S3 Fig 12 data. (XLSX)
