# Correction: Optimization design and experiment of key components of mountain pendulum-lever cam type hole seeders based on DEM-MBD coupling simulation

**DOI:** 10.1371/journal.pone.0343129

**Published:** 2026-02-17

**Authors:** Lijun Zhao, Wenke Yin, Bin Yang, Xin Hu, Chuandong Liu, Zihuan Li, Lian Gong, Qiang Li, Bin Li, Shang Li

There is an error in affiliation 1 for authors Lijun Zhao, Wenke Yin, Xin Hu, Chuandong Liu, Zihuan Li, Lian Gong, Qiang Li and Bin Li. The correct affiliation 1 is: College of Intelligent and Manufacturing Engineering, Chongqing University of Arts and Sciences, Chongqing, China.
