# Supplementary figures and images for: Optimization design and experiment of key components of mountain pendulum-lever cam type hole seeders based on DEM-MBD coupling simulation
Source: PLoS One. 2025 Mar 14;20(3):e0313285. doi: 10.1371/journal.pone.0313285 (PMC11908703; doi:10.1371/journal.pone.0313285)

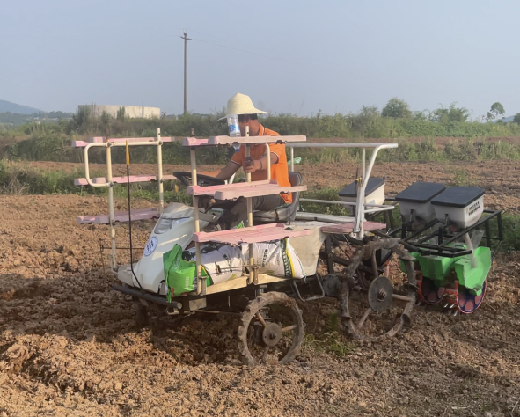


**Fig 16. Field trial.(a)**


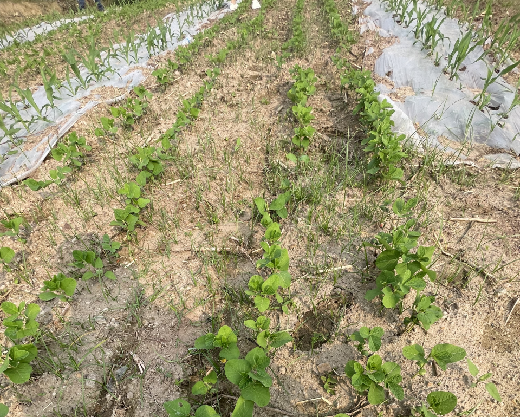


**Fig 16. Field trial.(b)**

Supplement: S1 Fig — (DOCX) [file pone.0313285.s001.docx]
