# Supplementary material for: Optimization design and experiment of key components of mountain pendulum-lever cam type hole seeders based on DEM-MBD coupling simulation
Source: PLoS One. 2025 Mar 14;20(3):e0313285. doi: 10.1371/journal.pone.0313285 (PMC11908703; doi:10.1371/journal.pone.0313285)
Supplement: S2 Fig — (DOCX) [file pone.0313285.s002.docx]

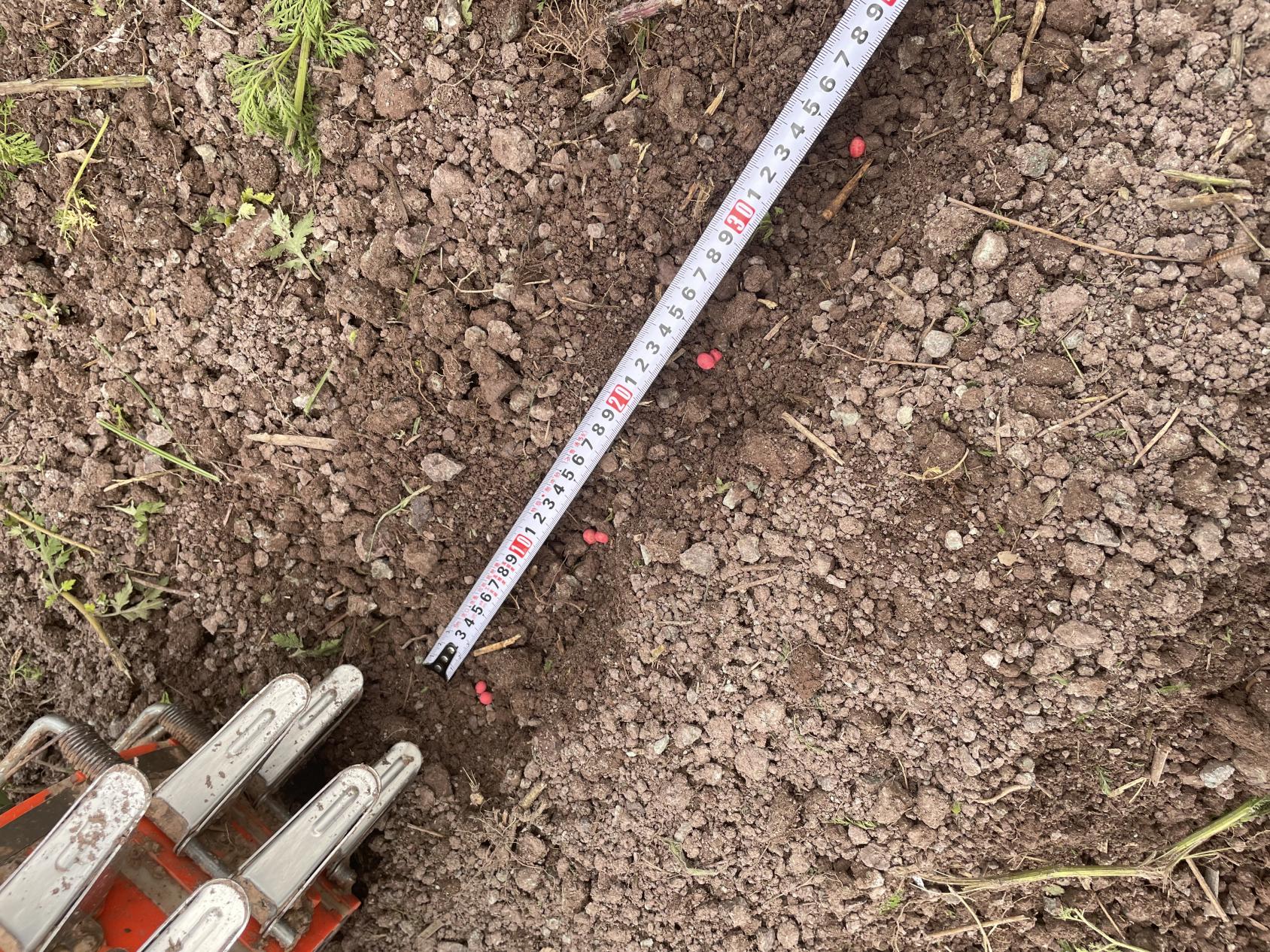


**Fig 17. Test results before and after optimization.(a)**

**
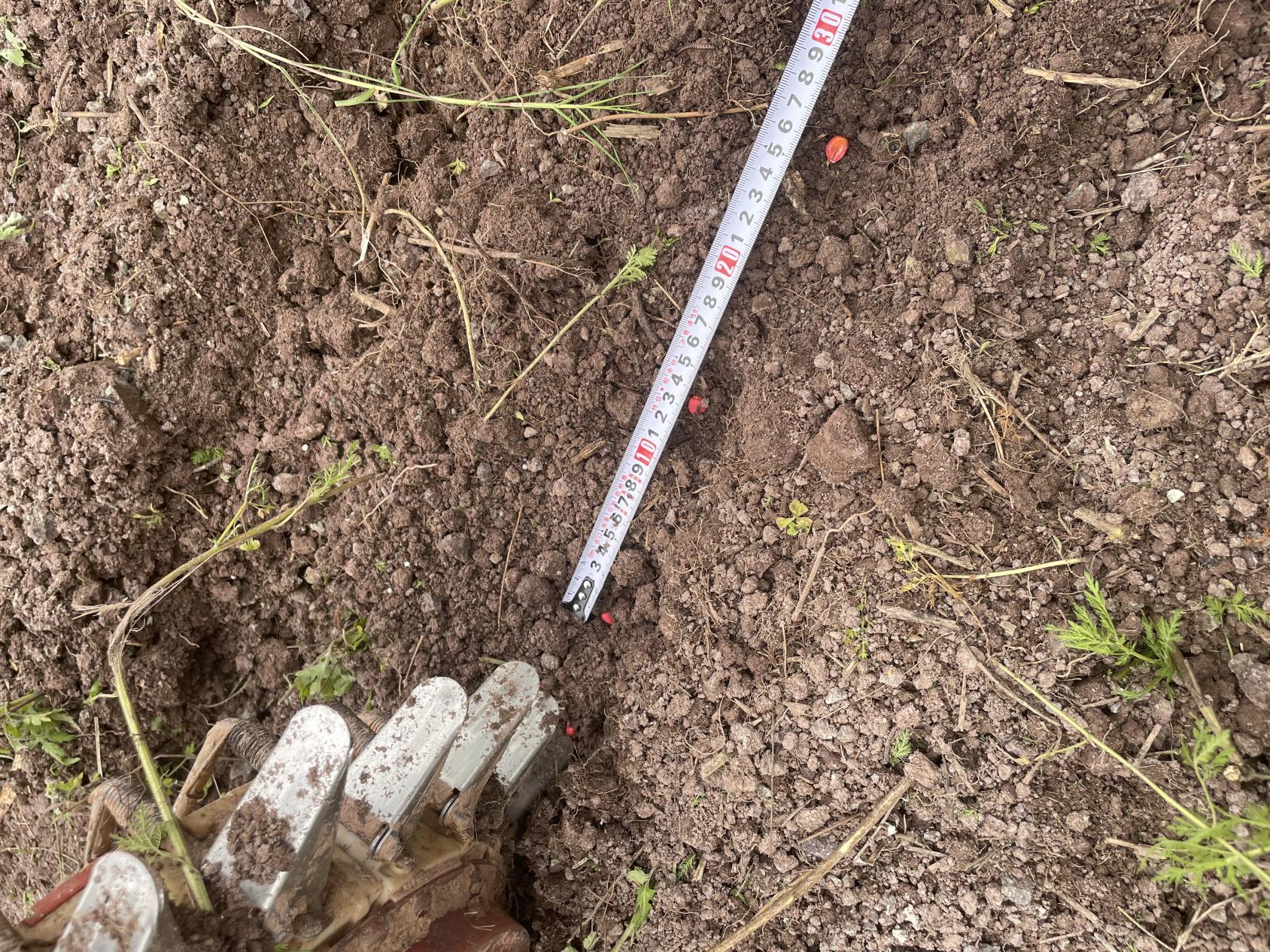
**

**Fig 17. Test results before and after optimization.(b)**

**
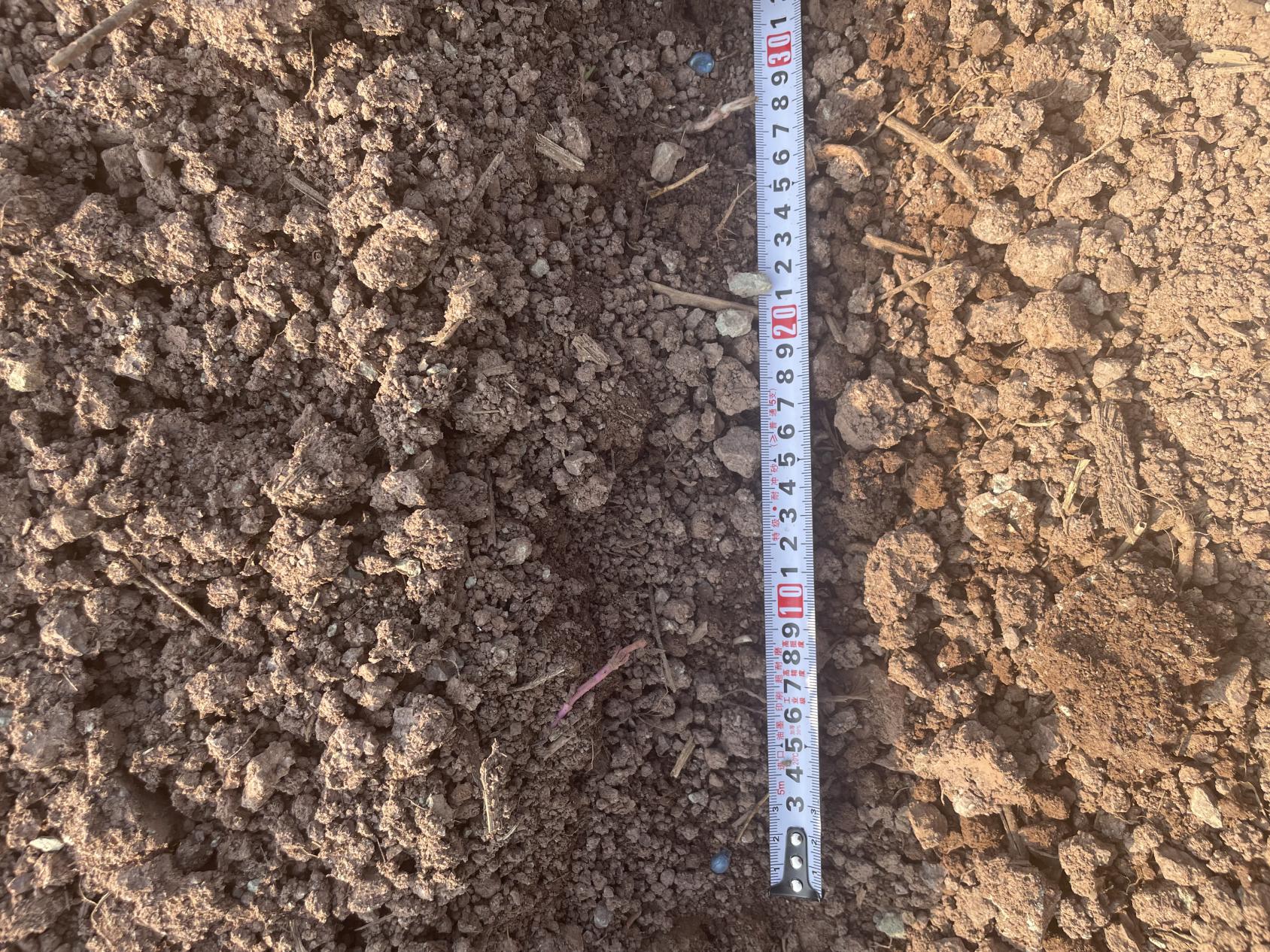
**

**Fig 17. Test results before and after optimization.(c)**

**
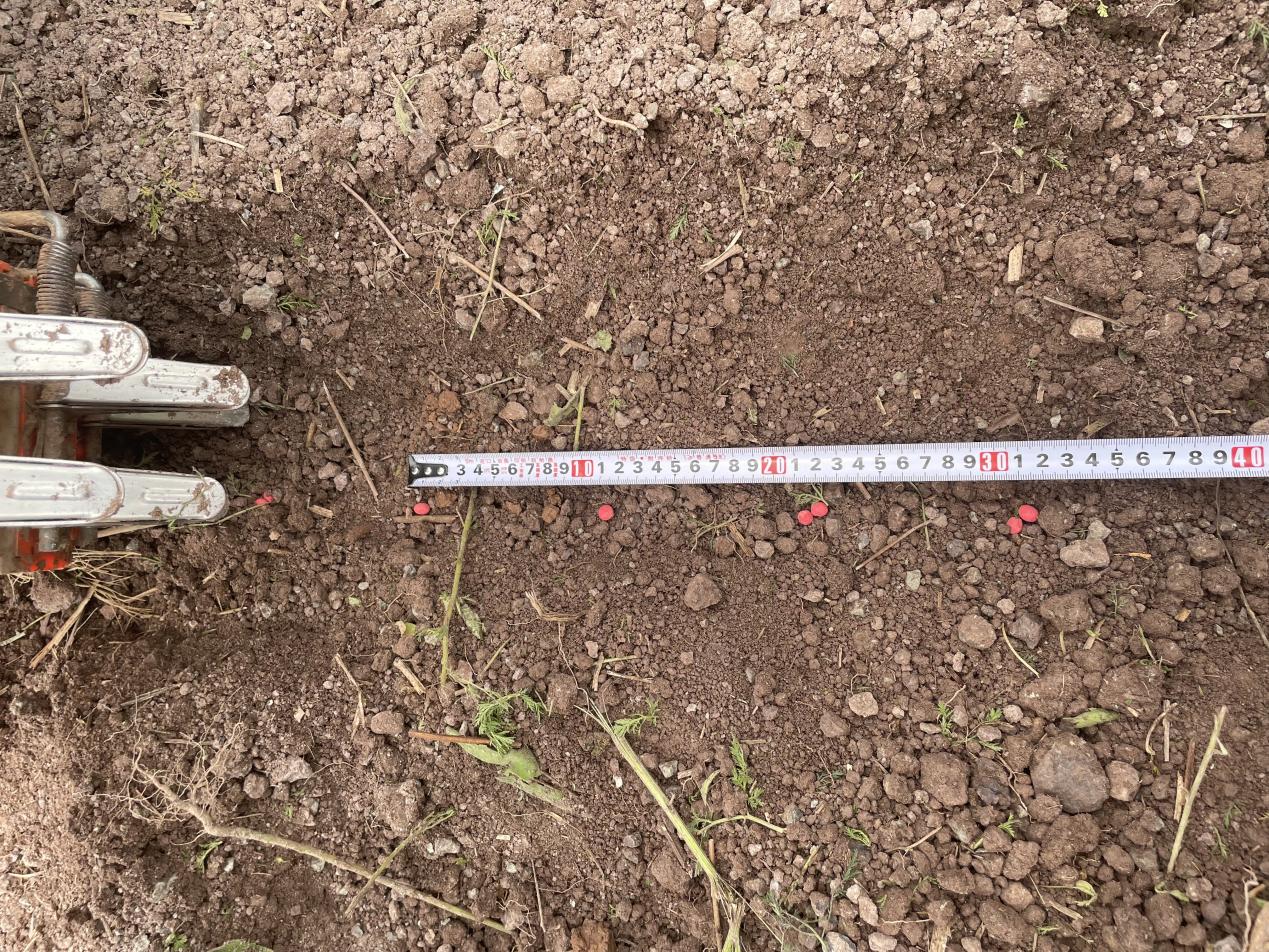
**

**Fig 17. Test results before and after optimization.(d)**

**
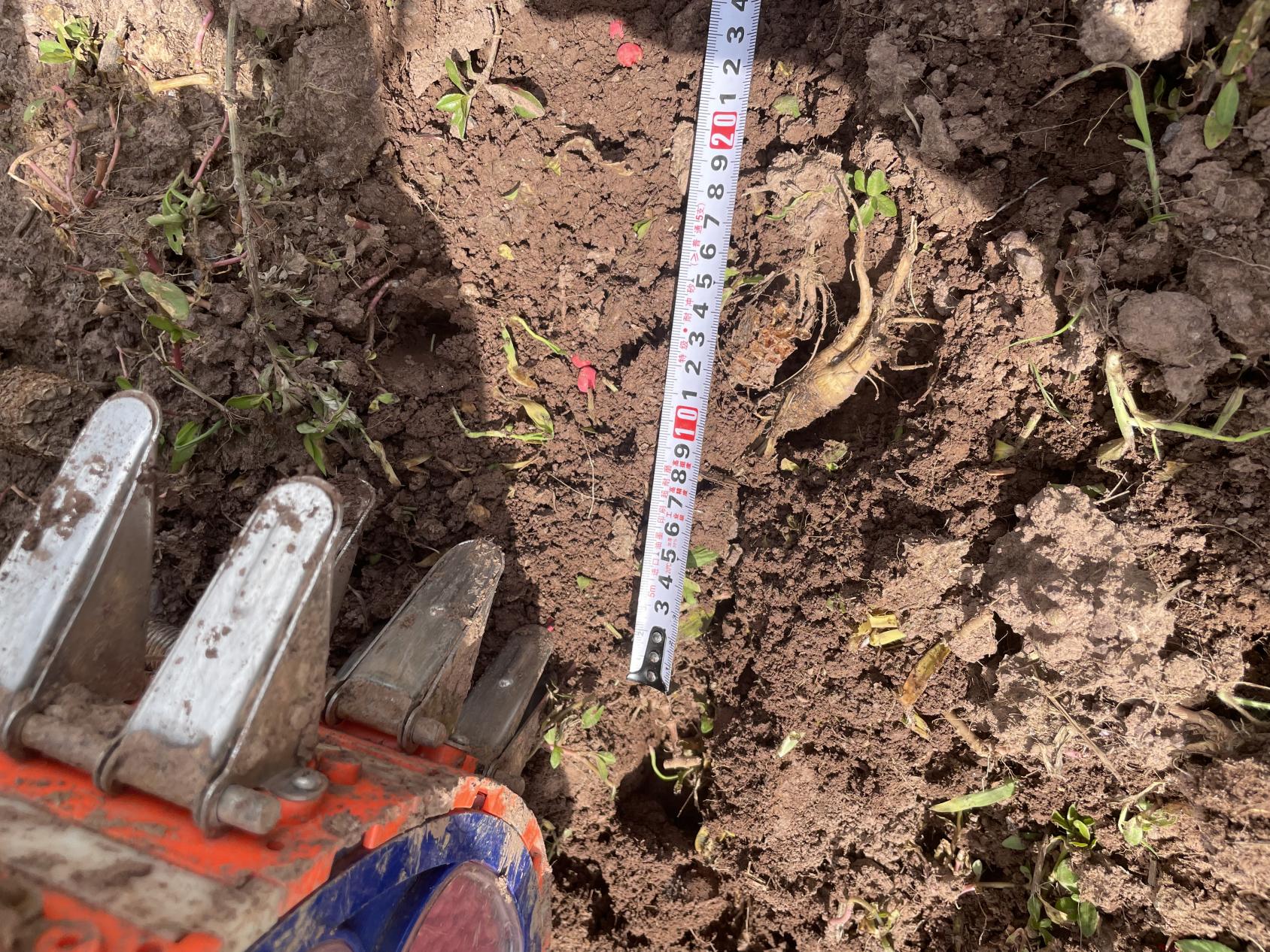
**

**Fig 17. Test results before and after optimization.(e)**

**
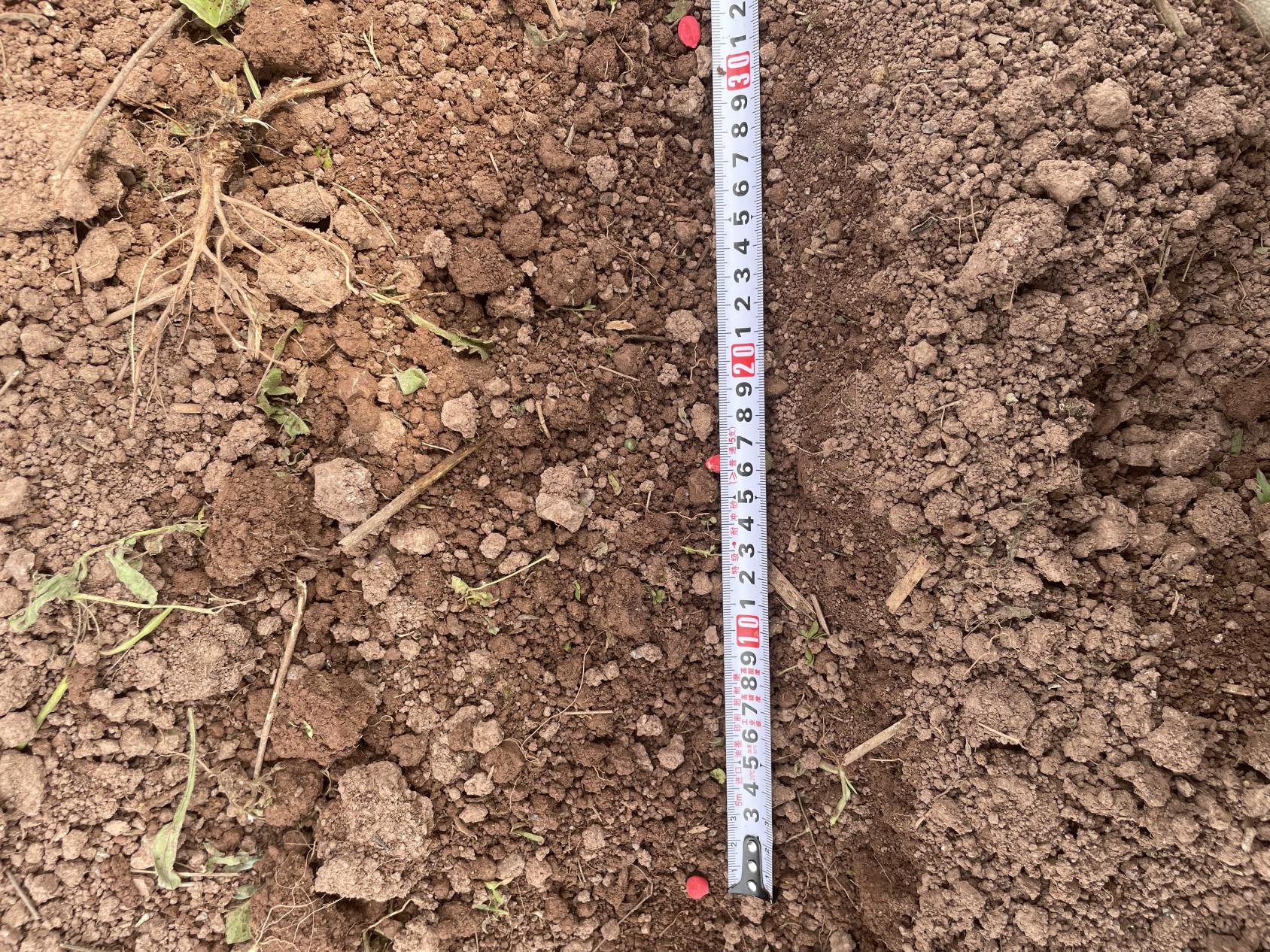
**

**Fig 17. Test results before and after optimization.(f)**
